# Unraveling attributes of COVID-19 vaccine acceptance and uptake in the U.S.: a large nationwide study

**DOI:** 10.1038/s41598-023-34340-3

**Published:** 2023-05-24

**Authors:** Sean D. McCabe, E. Adrianne Hammershaimb, David Cheng, Andy Shi, Derek Shyr, Shuting Shen, Lyndsey D. Cole, Jessica R. Cataldi, William Allen, Ryan Probasco, Ben Silbermann, Feng Zhang, Regan Marsh, Mark A. Travassos, Xihong Lin

**Affiliations:** 1 The How We Feel Project, San Leandro, CA USA; 2 Department of Biostatistics, Harvard T.H. Chan School of Public Health, Boston, MA USA; 3 Center for Vaccine Development and Global Health, University of Maryland School of Medicine, Baltimore, MD USA; 4 Department of Pediatrics, University of Maryland School of Medicine, Baltimore, MD USA; 5 Department of Pediatrics, University of Colorado Anschutz Medical Campus, Aurora, USA; 6 Adult and Child Consortium for Health Outcomes Research and Delivery Science, University of Colorado Anschutz Medical Campus and Children’s Hospital Colorado, Aurora, CO USA; 7 Harvard University, Cambridge, MA USA; 8 Department of Biological Engineering, Massachusetts Institute of Technology, Cambridge, MA USA; 9 McGovern Institute for Brain Research, Massachusetts Institute of Technology, Cambridge, MA USA; 10 Department of Brain and Cognitive Sciences, Massachusetts Institute of Technology, Cambridge, MA USA; 11 Howard Hughes Medical Institute, Chevy Chase, MD USA; 12 Department of Emergency Medicine, Brigham and Women’s Hospital, Boston, MA USA; 13 Department of Emergency Medicine, Harvard Medical School, Boston, MA USA; 14 Partners in Health, Boston, MA USA; 15 Broad Institute of MIT and Harvard, Cambridge, MA USA; 16 Department of Statistics, Harvard University, Cambridge, MA USA

**Keywords:** Public health, Infectious diseases, Disease prevention

## Abstract

SARS-CoV-2 vaccines are useful tools to combat the Coronavirus Disease 2019 (COVID-19) pandemic, but vaccine reluctance threatens these vaccines’ effectiveness. To address COVID-19 vaccine reluctance and ensure equitable distribution, understanding the extent of and factors associated with vaccine acceptance and uptake is critical. We report the results of a large nationwide study in the US conducted December 2020-May 2021 of 36,711 users from COVID-19-focused smartphone-based app How We Feel on their willingness to receive a COVID-19 vaccine. We identified sociodemographic and behavioral factors that were associated with COVID-19 vaccine acceptance and uptake, and we found several vulnerable groups at increased risk of COVID-19 burden, morbidity, and mortality were more likely to be reluctant to accept a vaccine and had lower rates of vaccination. Our findings highlight specific populations in which targeted efforts to develop education and outreach programs are needed to overcome poor vaccine acceptance and improve equitable access, diversity, and inclusion in the national response to COVID-19.

## Introduction

The emergence in late 2019 of severe acute respiratory syndrome coronavirus 2 (SARS-CoV-2) as a novel human pathogen and causative agent of the global coronavirus disease 2019 (COVID-19) pandemic^1^ fueled an unprecedented effort to rapidly develop a vaccine^2^. While the successful development of several effective SARS-CoV-2 vaccines was a major achievement, the defining challenge of the COVID-19 pandemic is ensuring equitable vaccine distribution and high vaccine uptake.

Soon after the identification of the virus, it was estimated that at least 70% of the U.S. population would need to acquire immunity to SARS-CoV-2 to end the COVID-19 pandemic^3^. It was unclear whether natural infection alone would produce sufficient, durable immunity, and vaccination became a major pillar of the public health strategy to control the pandemic. Public opinion polling in early 2020 suggested that as many as 72% of U.S. adults were willing to receive a COVID-19 vaccine once licensed and available. Four months later, the number of U.S. adults willing to receive a SARS-CoV-2 vaccine had sharply declined to as low as 51%^4^. Resistance to vaccination has posed a public health challenge since the smallpox vaccine was first invented, and although the vaccine targets and the cultural context may vary over time and place, common factors associated with reluctance, refusal, and even anti-vaccination activism include mistrust, misinformation, and a belief in the primacy of individual liberty.

In December 2020, two vaccines against COVID-19 received Emergency Use Authorization (EUA) from the U.S. Food and Drug Administration^5,6^. The results of phase 3 clinical trials and the subsequent rollout of the Pfizer-BioNTech and Moderna vaccines received significant attention in the media. Opinion polls conducted in December 2020 suggested a subsequent increase in public willingness to receive a COVID-19 vaccine, likely due to the widespread availability of data showing the vaccines to be both safe and effective^7^. Despite Johnson & Johnson’s Janssen COVID-19 vaccine also receiving EUA, national uptake of vaccines declined from mid-April 2021 onward as those reluctant to be vaccinated occupied a greater percentage of the unvaccinated population and information emerged about rare vaccine-related adverse events^8–10^.

How We Feel (HWF) is a web and mobile-phone application developed to facilitate the large-scale collection of data about COVID-19 symptoms, SARS-CoV-2 test results, and transmission-mitigating behaviors and sentiments^11^. Users are assigned a randomly generated number that tracks logins from the same device and are otherwise unidentifiable. Beginning in December 2020, we fielded a question about users’ COVID-19 vaccine intentions. These responses were then related to the user’s subsequent COVID-19 vaccine uptake or refusal.

We hypothesized that responses could provide significant insights into understanding vaccine acceptance across the United States, identifying populations that could be a promising focus of vaccine outreach efforts. We aimed to evaluate associations of the degree of COVID-19 vaccine acceptance in the U.S. and identify characteristics that might influence vaccine acceptance and eventual COVID-19 vaccine uptake. This has the potential to help public health and community leaders develop effective education and outreach programs to overcome vaccine reluctance and ensure equitable vaccine distribution and improved vaccine uptake.

## Results

A total of 36,711 users responded to the vaccine acceptance question. The largest number of respondents came from Connecticut and California with 8697 and 4668, respectively (Supplementary Fig. 1a). HWF’s user base is approximately 79% female (Supplementary Fig. 1b) and 83% white (Supplementary Fig. 1c). Users are 18 years of age or older and are equally distributed by age groups (Supplementary Fig. 1d). More than 68% of respondents were non-essential workers, and users cover a diverse range of income groups. All descriptive statistics of the study participants are available in Supplementary Table 1.

**Figure 1:**
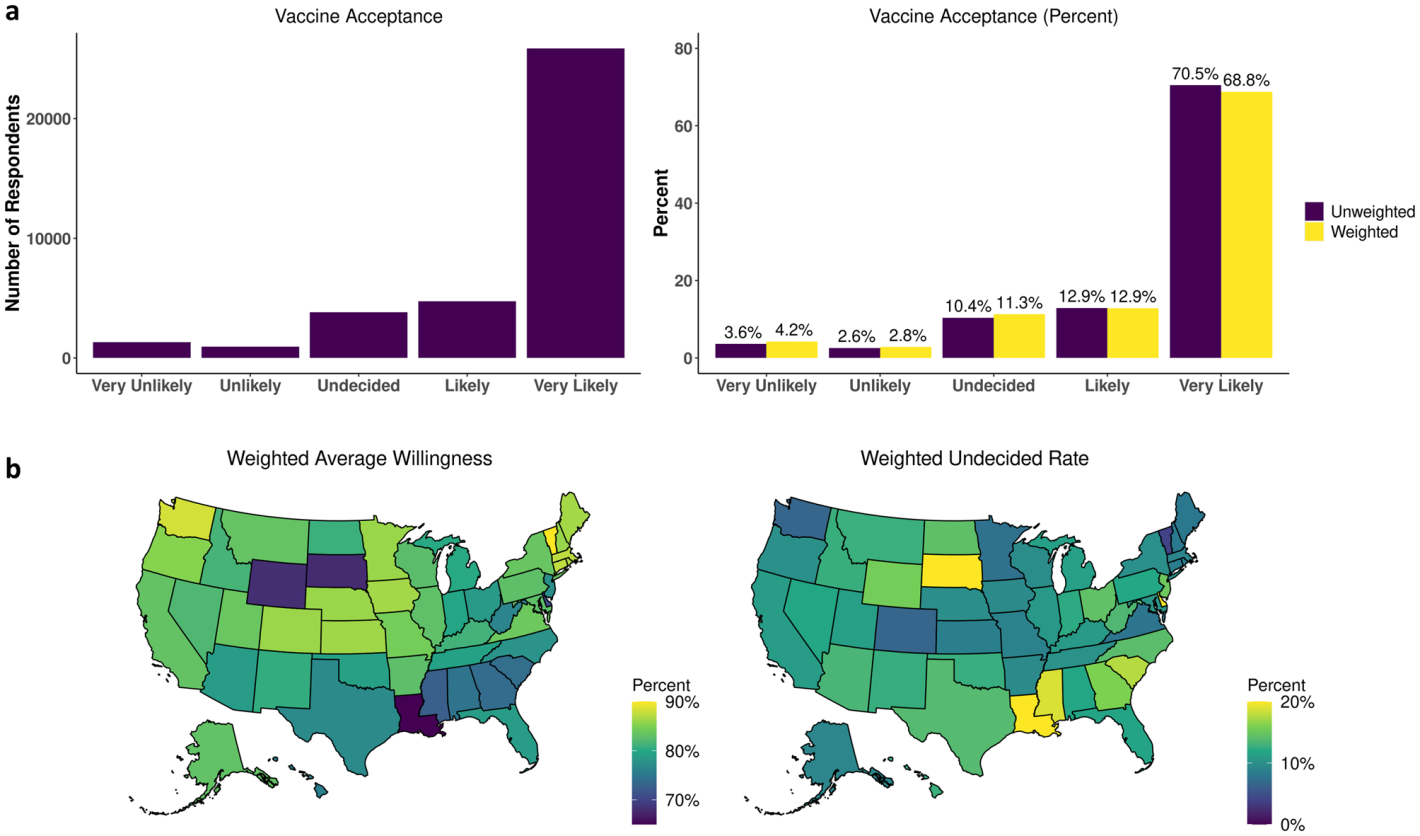
COVID-19 Acceptance rates: (**a**) (Left) Number of responses and (Right) unweighted and weighted percentages. (**b**) Weighted average willingness and undecided rates by state.

**Figure 2:**
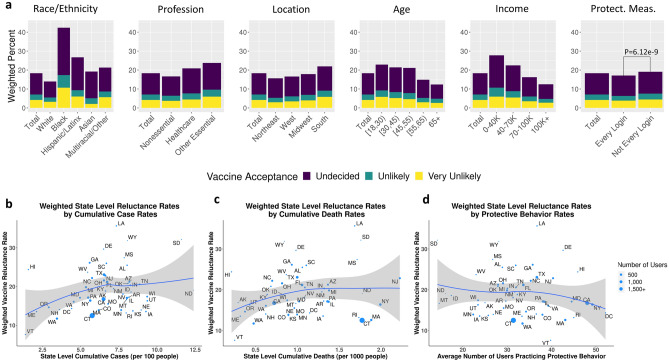
Demographic Acceptance Rates: (**a**) Weighted percentages of reluctant responses by race/ethnicity, profession, location, age, income, and use of protective measures. State level weighted reluctance rates by (**b**) cumulative case rates (/100 individuals), (**c**) cumulative death rates (/1000 individuals), (**d**) and average number of users practicing protective behavior.

In total, 30,618 (83%) were willing (“Likely” or “Very Likely”) to be vaccinated (Fig. 1a). After applying a census-based post-stratification weight (see Methods), Vermont (92%) and Washington D.C. (88%) had the highest rates of vaccine reluctance while South Dakota (27%) and Louisiana (23%) had the highest rates of undecided users (Fig. 1b). Weighted bar plots of vaccine reluctance across demographic characteristics revealed that “Undecided” users represented the largest proportion of non-willing users across all demographic groups (Fig. 2a, Supplementary Table 2).

State level reluctance (“Undecided”, “Unlikely”, or “Very Unlikely”) rates were negatively associated with the average number of users that practiced transmission mitigating behaviors and were positively associated with cumulative COVID-19 case and death rates by January 10, 2021 (Fig. 2b–d). Unweighted plots are available in Supplementary Fig. 2.

**Figure 3:**
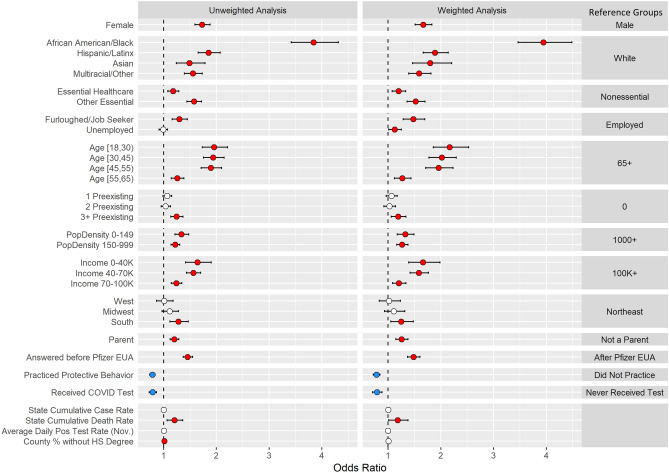
Logistic regression-based association analysis results of vaccine acceptance: Forest plots for (Left) unweighted and (Right) weighted multivariable logistic regression analyses for vaccine reluctance with 95% confidence intervals. Non-significant variables at the 0.05 level (white), significant positive associations (red), and significant negative associations (blue).

To assess demographic associations with vaccine acceptance, we fit a univariate logistic regression with socio-demographic, occupation, preexisting medical conditions, geographical and COVID-19 related predictors (Supplementary Table 3) and a multivariable logistic regression model to adjust for potential relationships between the predictors (Fig. 3, Supplementary Table 4). We implemented post-stratification weights using census estimates of sex, age, race, and census location (see Methods). People of color reported higher rates of vaccine reluctance compared to white non-Hispanic users (African American OR, 3.94; CI, 3.47, 4.48; *p* = 1.26e−96). Vaccine reluctance was more likely among females than males (OR,1.67; CI, 1.51, 1.83; *p* = 4.09e−25); younger users than those over 65 years old (18–30 OR, 2.17; CI, 1.86, 2.53; *p* = 1.03e−22); those with three or more preexisting conditions than those with zero (OR, 1.19; CI, 1.06, 1.34; *p* = 0.0036); and parents than non-parents (OR, 1.26; CI, 1.15, 1.38; *p* = 9.61e−7). Individuals that were furloughed or job-seeking were also more vaccine reluctant compared to those working full- or part-time (OR, 1.48; CI, 1.29, 1.70; *p* = 4.04e−8). Respondents from the South (OR, 1.25; CI, 1.05, 1.48; *p* = 0.0105), from less densely populated areas, or with lower incomes were all more likely to be vaccine reluctant. Users that responded before the Pfizer Emergency Use Authorization (EUA) on December 11, 2020 were more vaccine reluctant than users who responded after the Pfizer EUA (OR, 1.48; CI, 1.37, 1.60; *p* = 9.96e−23), users who practiced behavior protective against COVID-19 such as mask-wearing or social distancing were less vaccine reluctant (OR, 0.78; CI, 0.72, 0.85; *p* = 6.12e−9), and users that received a COVID test were less vaccine reluctant (OR, 0.79; CI, 0.71, 0.89, *p* = 5.88e-5).

Nominal logistic regression (see Methods) evaluated whether vaccine reluctance was driven by “Undecided” vs. “Unlikely/Very Unlikely” responses (Supplementary Table 5) and was also conducted with a weighted analysis (Supplementary Table 6). Reluctance in healthcare workers, those aged 55–64, Asian users, and those in locations with a median income between $70,000 and $100,000 was driven by the “Undecided” group, whereas reluctance in the unemployed, those with 3 + preexisting conditions, and southern users was driven by the “Unlikely” group. Sensitivity analyses were performed for the weighted multivariable and nominal regression analyses with a less restrictive threshold for the trimming weights (Supplementary Table 7–8) and found similar results. We conducted a sensitivity analysis to assess differences in reluctance in individuals that tested positive for COVID-19 and found no difference in intention based on testing results (see Methods, Supplementary Table 9–10).

**Figure 4:**
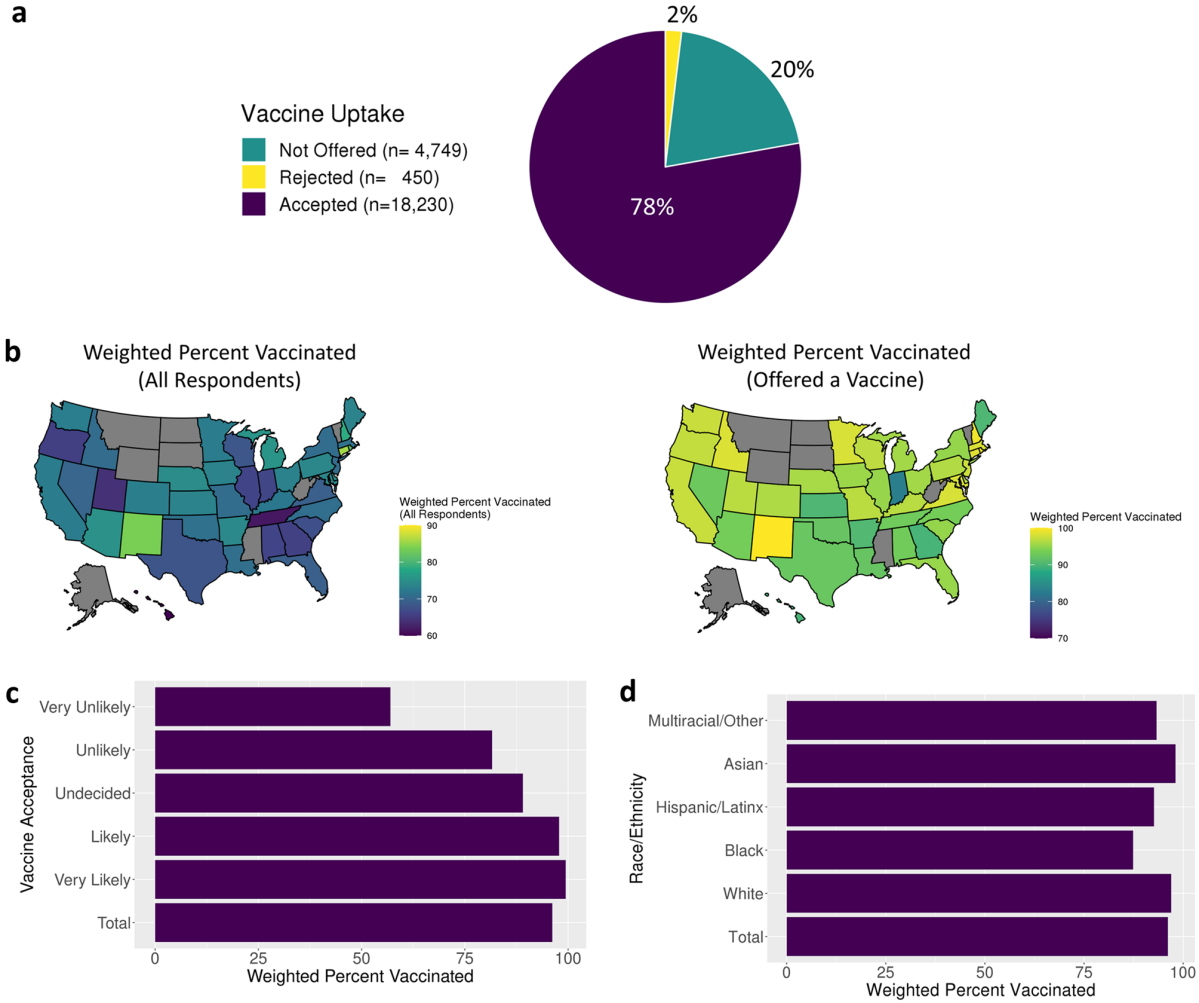
Vaccine Uptake Rates: (**a**) Vaccine uptake question responses for all users. (**b**) Weighted vaccination rates by state of (Left) all users that responded to the vaccine uptake question and (right) users that were offered a vaccine. (**c**) Weighted vaccination uptake of users that were offered a vaccine by vaccine acceptance and (**d**) race/ethnicity.

Of the 36,711 users who responded to the vaccine acceptance question, 23,429 also responded to the vaccine uptake question and its distribution is provided in Fig. 4a. Demographic distributions remained similar to those of respondents of the vaccine acceptance question with a slight increase in the proportion of users ages 65 + (Supplementary Fig. 3). Vaccination rates by state are shown in Fig. 4b for all users that responded to the vaccine uptake question and subset to respondents who were offered a vaccine. Users with lower levels of vaccine acceptance had lower rates of vaccination (Fig. 4c), and Black and Hispanic/Latinx users reported lower rates of vaccination than White, Non-Hispanic users (Fig. 4d). Plots of weighted and unweighted vaccination rates across all demographic features are available in Supplementary Figs. 4–5.

**Figure 5:**
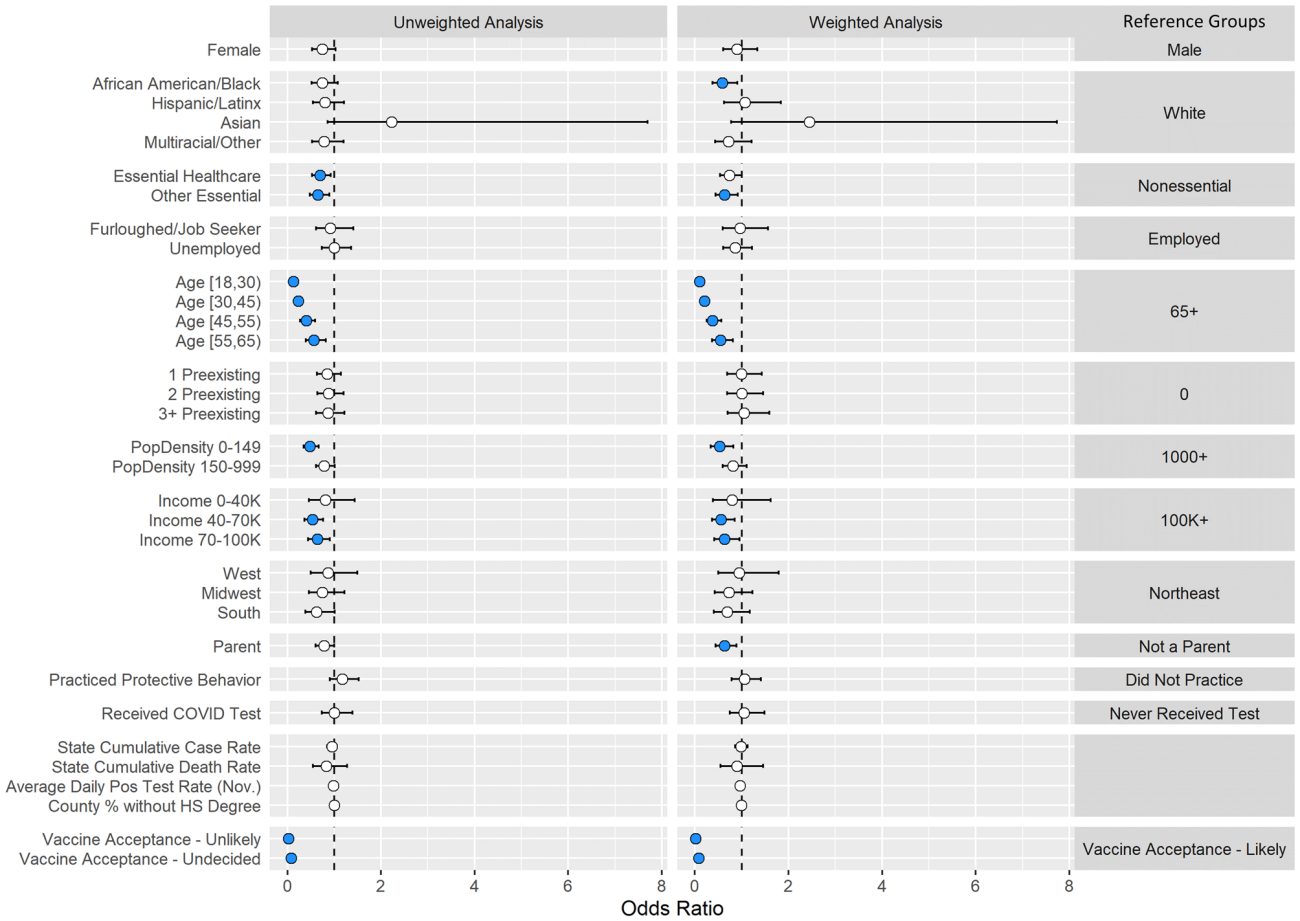
Logistic regression-based association analysis results of vaccine uptake: Forest plots for (Left) unweighted and (Right) weighted multivariable logistic regression analyses for vaccination uptake with 95% confidence intervals. Non-significant variables at the 0.05 level (white), significant positive associations (red), and significant negative associations (blue).

To formally identify demographic features associated with differences in vaccination rates, we conducted an unweighted and weighted multiple logistic regression analysis (see Methods, Fig. 5, Supplementary Tables 11–12). All age groups reported lower rates of vaccinations compared to users over 65 (18–30 OR: 0.10; CI, 0.06, 0.18; *p* = 1.43e−16); Black users reported lower rates of vaccinations (OR, 0.58; CI, 0.38, 0.91; *p* = 0.0165) compared to White non-Hispanic users; essential workers outside of healthcare reported lower rates of vaccinations (OR, 0.64; CI, 0.44, 0.92; *p* = 0.0162) compared to non-essential workers; parents reported lower rates of vaccination (OR, 0.63; CI, 0.45, 0.89; *p* = 0.0086) compared to users who are not parents; users in areas with a median household income (MHI) of $40–70 K (OR, 0.56; CI, 0.37, 0.85; *p* = 0.0066) and $70–100 K (OR, 0.63; CI, 0.42, 0.96; *p* = 0.0316) reported lower rates of vaccinations compared to those in areas with a MHI $100 K + ; users logging in from areas with 0–149 people/sq. mi reported lower rates of vaccinations (OR, 0.53; CI, 0.34, 0.82; *p* = 0.0049) compared to users in high population density areas; and users that responded “Unlikely/Very Unlikely” (OR, 0.02; CI, 0.01, 0.03; *p* = 2.07e−114) and “Undecided” (OR, 0.08; CI, 0.06, 0.12; *p* = 1.06e−39) to the vaccine acceptance question reported lower rates of vaccinations compared to willing users.

While vaccination rates were lower in the reluctant group compared to the acceptant group, 86% (2157/2520) of reluctant users were vaccinated. In a formal multiple regression analysis looking at demographic associations with vaccine uptake among reluctant users, similar associations were found (see Methods, Supplementary Table 13). Younger age groups, healthcare workers, people from lower income households, and residents of areas with lower population density had lower vaccination rates. Users who responded to the vaccine acceptance question as “Undecided” reported higher rates of vaccination compared to those that responded “Unlikely/Very Unlikely” (OR, 4.57; CI, 3.47, 6.03; *p* = 2.26e−26).

## Discussion

In our analysis, increased reluctance was associated with minority race/ethnicity, living in less densely populated regions, and being a healthcare worker. A large proportion of these populations were undecided about COVID-19 vaccination, suggesting that targeted outreach may improve vaccine uptake. In fact, a significant portion of those skeptical or undecided about vaccination were ultimately vaccinated, supporting the idea that perspectives on COVID-19 vaccination are not immutable and may respond to such outreach.

Black respondents had the highest rates of COVID-19 vaccine reluctance and the lowest rates of vaccine uptake relative to other racial and ethnic groups, consistent with other surveys^4,9,10,12–15^. The history of racist practices within the U.S. healthcare system and research community, such as during the Tuskegee Syphilis Study^16^, and disparities in social determinants of health including poor access to healthcare and limited time off work likely contribute to our findings. Dispelling concerns within the Black community requires extensive, sustained, structured outreach and will be critical to efforts to contain and eliminate COVID-19. The National Institutes of Health’s Community Engagement Alliance (CEAL) provides a model for such outreach, targeting populations that have been hardest hit by the COVID-19 pandemic^17^.

Education and outreach efforts must target several additional populations. This includes rural residents and young adults. Because large proportions of these populations were undecided about COVID-19 vaccination, outreach to these groups must also provide reliable vaccine information tailored to the needs of each community, and different outreach strategies may be needed to address the concerns of those who were undecided and those who were unlikely.

Vaccine acceptance in healthcare workers warrants particular attention. We found that reluctant healthcare workers were less likely than other reluctant workers to change their mind (Supplementary Table 13). Others have found that U.S. nurses had the highest degree of COVID-19 vaccine reluctance among healthcare workers^18^. As the profession that enjoys the highest degree of public trust, nurses have an important role to play in promoting vaccine confidence^19^. Furthermore, inadequate vaccine uptake among healthcare workers raises the possibility of sustained COVID-19 transmission in an essential worker population critical to caring for vulnerable members of society, including immunocompromised individuals and children, the majority of whom were not yet eligible for a COVID-19 vaccine by the conclusion of this study^20–23^.

Addressing regional foci of reluctance to accept a COVID-19 vaccine will be critical in federal resource allocation to combat vaccine reluctance in general. We identified the greatest level of reluctance to accept a COVID-19 vaccine in the South followed by the Midwest. While a survey sponsored by the United States Centers for Disease Control and Prevention (CDC) and conducted in December 2020 found that COVID-19 vaccine hesitancy was most prevalent in the Northeast, followed by the South^15^, other data from the CDC detailing U.S. state and county-level vaccination rates and allocated dose usage have consistently shown that Southern states have lower vaccination rates and lower allocated dose usages compared to other areas of the country^9^. The significance of these phenomena is highlighted by the resurgence of COVID-19 with the spread of the delta variant in the South^24^.

Initial reluctance or indecision regarding COVID-19 vaccination was not fixed and did not necessarily reflect a respondent's eventual vaccination decision. This suggests the need for a multi-pronged approach that includes interventions directed at behavior change. Even if receptivity towards vaccination is low, there may still be significant potential for increasing vaccine uptake, indicating the need for continued implementation of strategies known to be effective, such as health care provider outreach and reminders^25,26^.

A study limitation is that our sample may not be generalizable to the broader American public or to populations outside of the U.S., particularly lower- and middle-income countries. How We Feel users are self-selecting, technologically literate, and more likely to have a high baseline level of concern about COVID-19. The user base is inherently skewed by a large proportion of users residing in Connecticut and California and by regional age discrepancies. Given the Connecticut government’s involvement in promoting the application, it’s possible users from Connecticut are more trusting of their state’s government. Census-adjusted, weighted analysis help correct the sampling bias but may not completely remove the potential for bias, and interpretation of our findings should note this. Furthermore, interstate movement of respondents during the pandemic may have affected the geographic distribution of responses. Additionally, we were unable to objectively verify self-reported vaccination; however, in other independent studies, there was a high degree of concordance between self-reported influenza vaccination and respondents’ actual influenza vaccination status^27,28^. This provides indirect evidence that self-reported COVID-19 vaccination status is a good proxy of verified vaccination status. Future research needs to be conducted to verify the concordance between the self-reported and registry-based vaccination records.

Further work is needed to better understand how vaccine reluctance relates to novel vaccine uptake in the U.S. and to understand how knowledge, attitudes, and behaviors surrounding COVID-19 vaccines change over time. As COVID-19 vaccines have become widely available to adults and adolescents in the U.S. and COVID-19 restrictions are lifting, our findings affirm the ongoing need to address vaccine reluctance and issues related to access.

## Methods

### Open-source software

We used the following open-source software in the analysis.

- R: http://www.r-project.org
- Tidyverse: http://www.tidyverse.org
- Data.table: https://CRAN.R-project.org/package=data.table
- nnet https://CRAN.R-project.org/package=nnet
- censusapi https://CRAN.R-project.org/package=censusapi
- survey https://CRAN.R-project.org/package=survey
- ggplot2 https://CRAN.R-project.org/package=ggplot2
- cowplot https://CRAN.R-project.org/package=cowplot

### Data collection

Users could freely download the application which was available for both Android and Apple devices. The application was advertised widely on various social media outlets and through a partnership with the Connecticut state government which provided press releases to encourage residents to download the application. Users also heard about the application through word of mouth and through general media coverage. Data on vaccine acceptance was collected between December 4th, 2020 and May 6th 2021^11^. Following guidance from the CDC, users were asked “If a safe, effective coronavirus vaccine were available, how likely would you be to get yourself vaccinated?” Responses were given on a bipolar 5-point Likert scale from “Very Unlikely” to “Very Likely”, with “Undecided” being the middle value. The users first recorded response to the vaccine acceptance question was used in this analysis. On February 12th, 2021, a vaccine uptake question was added. Users were asked “Have you received a COVID-19 vaccine?” and could respond with “Yes”, “No, I haven’t been offered one”, or “No, I have been offered one but declined”. For all uptake models the most recent response was used. A consort diagram is available in Supplementary Fig. 7 to further clarify the number of respondents.

Users also self-reported race/ethnicity, sex, age, occupation, and preexisting conditions. Users who identified as “other” in the gender response were dropped due to small sample size. Neighborhood specific median household income was obtained from the user’s zip code at the time of answering the vaccine acceptance question by using the American Community Survey 5-year average results from 2018. Population density was calculated at the county level for each user based on data from the Yu Group at University of California at Berkeley^29^. State level case and death rates were obtained from USAFACTS^30^. As a proxy for user’s education status, the percentage of residents without a high school degree was included for each user’s county from the Census database.

Race/ethnicity was defined using distinct groups corresponding to “White,” “Black/African-American,” “Hispanic/Latino,” and “Asian” if the user only selected that respective racial group. Users who answered more than one race or ethnicity or selected an option other than the ones listed above were placed in a “multiracial/other” category.

During each login, users reported whether they left their home and for what reason. If they left home, they were then asked what types of protective measurements they used while away (mask, social distancing, cloth mask, and/or avoiding public transportation). We defined “protective behavior” to be if a user either stayed home or wore a mask when outside the home. If the user said that they did not wear a mask outside the home but engaged only in outdoor exercise and maintained physical distance from others, then they were also considered to be practicing protective behavior. We then created a variable that was coded as “1” if they always practiced protective behavior during all logins and a “0” if they failed to be protective during at least one login.

### Modeling

Users were considered to be reluctant to accept a vaccine if they responded as “Very Unlikely,” “Unlikely,” or “Undecided” to the vaccine acceptance question. Using vaccine reluctance as the outcome, a logistic regression was fit using several demographic variables as predictors to identify characteristics of users that were more or less vaccine reluctant. Both a univariate (Supplementary Table 3) and a multivariable model (Fig. 3, Supplementary Table 4) were performed to adjust for potential confounding. Only responses from users residing within the United States were used in the modelling. Corresponding odds ratios and 95% confidence intervals are provided, and statistical significance was assessed at the 0.05 level. Analyses were conducted using R (v 3.5.1).

Using the same covariates as in the logistic regression, a nominal logistic regression was fit to assess if results from the logistic regression were driven by individuals being more likely to be in the “Undecided” or “Unlikely” groups. The 5-point Likert scale was reduced to a 3-level bipolar variable for modelling purposes by combining “Very Unlikely” with “Unlikely” and “Very Likely” with “Likely” (Supplementary Table 5).

### Weighted analysis

To adjust our analyses to a user base that matches the major U.S. census demographics, we implemented a weighted analysis using post-stratification weights. Using the census population estimates of sex, race, age, and census location, a population-based joint distribution was obtained. A user base distribution was also calculated using the same breakdown, and the two proportions were then matched per user. The post-stratification weight was then calculated by dividing the census proportion by the sample proportion plus 1e−4 to avoid issues with smaller user base probabilities. To avoid over or underweighting individuals, the post-stratification weights were trimmed to be between 0.3 and 3 prior to the weighted analysis (Supplementary Table 4). For the nominal regression analysis, two separate weighted logistic regressions were conducted. One compared the “Undecided” group vs. the “Likely” group, while the other compared the “Unlikely” group vs. the “Likely” group (Supplementary Table 6). To assess the choice of the weight trimming bounds, sensitivity analyses were conducted for both above weighted analyses (Supplementary Table 7–8) using a threshold of 0.1 and 5. Supplementary Fig. 6 provides the distribution of the post-stratification weights.

### IPW analysis

To formally assess if there was a difference in vaccine reluctance between those that received a prior positive COVID test and those that received a negative test, we adjust for the demographic biases associated with receiving a COVID test. We first fit a weighted logistic regression to model the probability of receiving a test using all individuals and all demographic features that have been reported in previous analyses while applying the same weighted procedure as above. The coefficients, 95% confidence intervals, and *p*-values for this analysis are available in Supplementary Table 9. The fitted probabilities were then used as inverse probability weights (IPWs) in a weighted logistic regression model for vaccine reluctance only including individuals which had received a COVID test. The same predictors for previous weighted models were used and a new variable designating if a user tested positive or negative was included. To avoid extreme high or low weights, the fitted probabilities were trimmed to be between 0.1 and 0.9 or 0.05 and 0.95. The results of both models are available in Supplementary Table 10.

### Vaccine uptake

An unweighted multivariable logistic regression model was fit to identify which demographic features were associated with accepting or rejecting a COVID-19 vaccine. Along with the covariates included in the vaccine intent model, the three-level vaccine acceptance variable (“Very Likely/Likely”, “Undecided”, “Very Unlikely/Unlikely”) was also included in the analysis. Results are available in Supplementary Table 12 (left). To account for the biased sampling, non-response bias, and demographic differences in being offered a vaccine, a weighted multivariable model was fit. First, a weighted multivariable logistic regression model was fit for the probability of an individual responding to the vaccine uptake question with the inclusion of post-stratification weights as was done in the weighted vaccine acceptance model (Supplementary Table 11 A). The fitted probabilities from this model were then used as inverse probability weights to model the probability of a user being offered a vaccine (Supplementary Table 11 B). A user was defined as being offered a vaccine if the user responded to the question “Have you received a COVID-19 vaccine?” with “Yes,” or “No, I have been offered one but declined,” compared to users responding “No, I have not been offered a vaccine.” The fitted probabilities from this model were multiplied by the fitted probabilities from the response model and used as inverse probability weights in a final model which models the probability of accepting or rejecting the vaccine. The coefficients, 95% confidence intervals, and *p*-values for the final weighted model are available in Supplementary Table 12. To more formally characterize the attributes associated with vaccine uptake within users that responded as vaccine reluctant, we fit a weighted multivariable logistic regression model subset to only the users who initially responded they were “Very Unlikely” or “Unlikely” to receive a COVID-19 vaccine. Models were fit identically to the above weighted models for all users and results of the final model are available in Supplementary Table 13.

### Ethics statement

Data was obtained from the non-profit organization the How We Feel Project which obtained a commercial IRB approval for the collection of the data. Due to receiving a deidentified dataset, the analysis in this paper was exempt from Institutional Review Board (IRB) approval by Harvard University Longwood Medical Area (HULC) IRB (HULC IRB Protocol No. IRB20-0514) and the Broad Institute of MIT and Harvard IRB (Broad/Harvard IRB Protocol no. EX-1653). When downloading the application, users were informed that their data would be shared securely with scientists, doctors, and public health professionals to stop the spread of COVID-19 and provided informd consent.

## Supplementary Information

Supplementary Information 1.

Supplementary Information 2.

## Data Availability

This work used data from the How We Feel project. The data are not publicly available, but researchers can apply to use the resource. Researchers with an appropriate IRB approval and data security approval to perform research involving human subjects using the How We Feel data can apply to obtain access to data used in the analysis.

## Code availability

The analysis code developed for this paper can be found online at https://github.com/mccabes292/HWF_VaccineHes_Paper.
