# Supplementary material for: Unraveling attributes of COVID-19 vaccine acceptance and uptake in the U.S.: a large nationwide study
Source: Sci Rep. 2023 May 24;13:8360. doi: 10.1038/s41598-023-34340-3 (PMC10209066; doi:10.1038/s41598-023-34340-3)
Supplement: Supplementary file 1 — Supplementary Information 1. [file 41598_2023_34340_MOESM1_ESM.pdf]

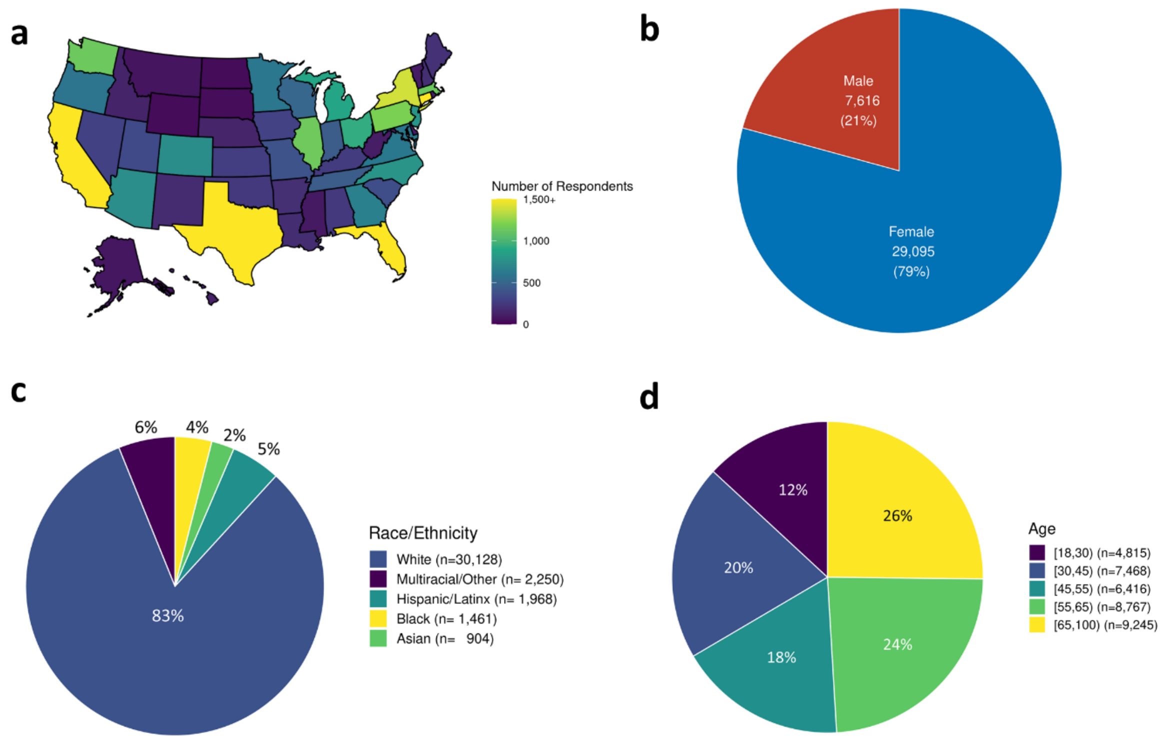

Supplementary Figure 1, HWF Vaccine Acceptance Demographic Distributions : Demographic break down of the HWF user base that responded to the vaccine acceptance question by **(a)** state, **(b)** sex, **(c)** race and **(d)** age.

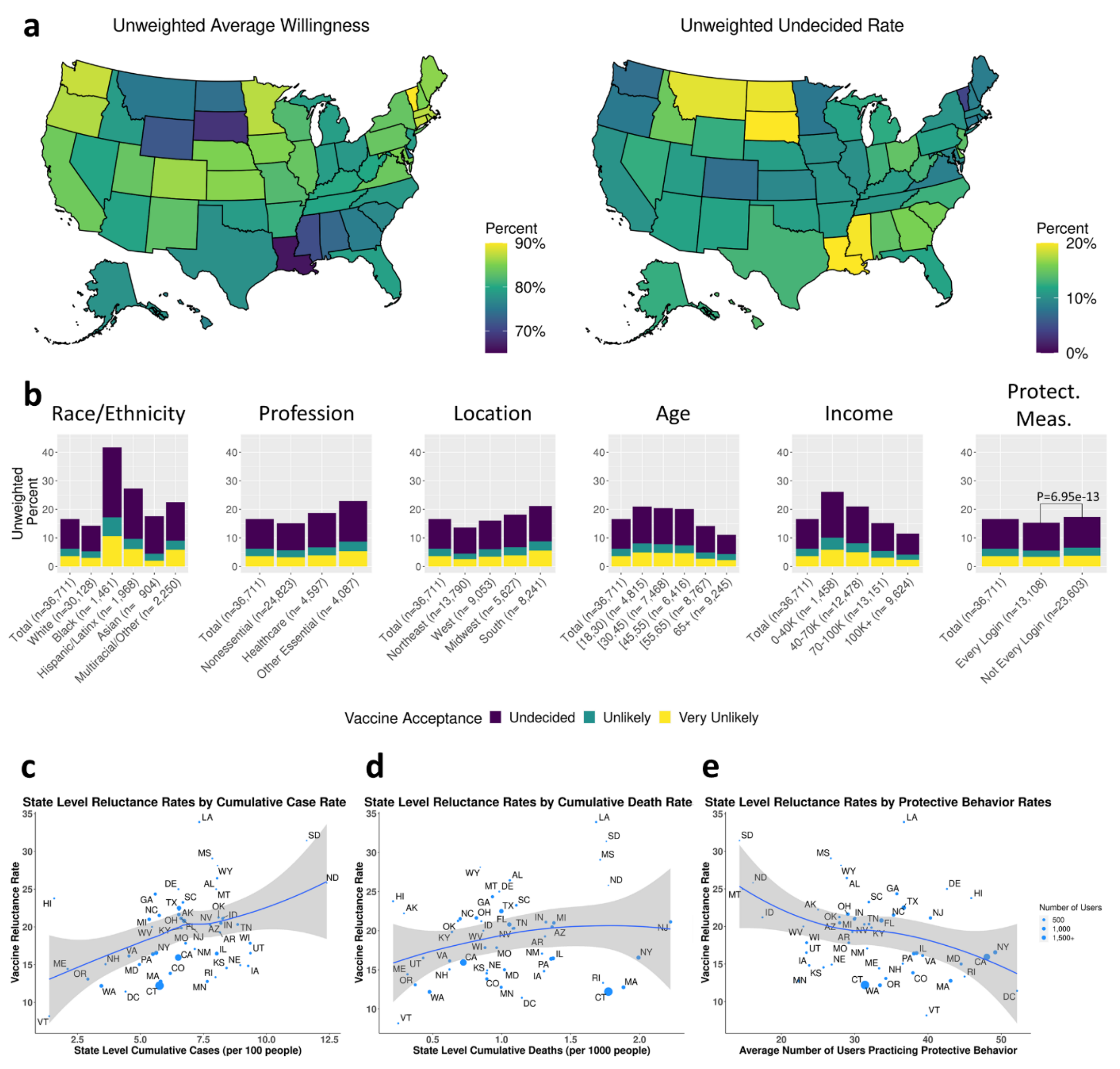

Supplementary Figure 2, Unweighted vaccine acceptance responses: (a) Unweighted statewide (Left) willingness and (Right) undecided rates. **(b)** Unweighted vaccine acceptance responses across several demographic characteristics. State level reluctance rates estimated using the How We Feel data by **(c)** cumulative case (per 100 individuals) and **(d)** death (per 1000 individuals) rates reported in USA Facts and **(e)** average number of users practicing protective behavior.

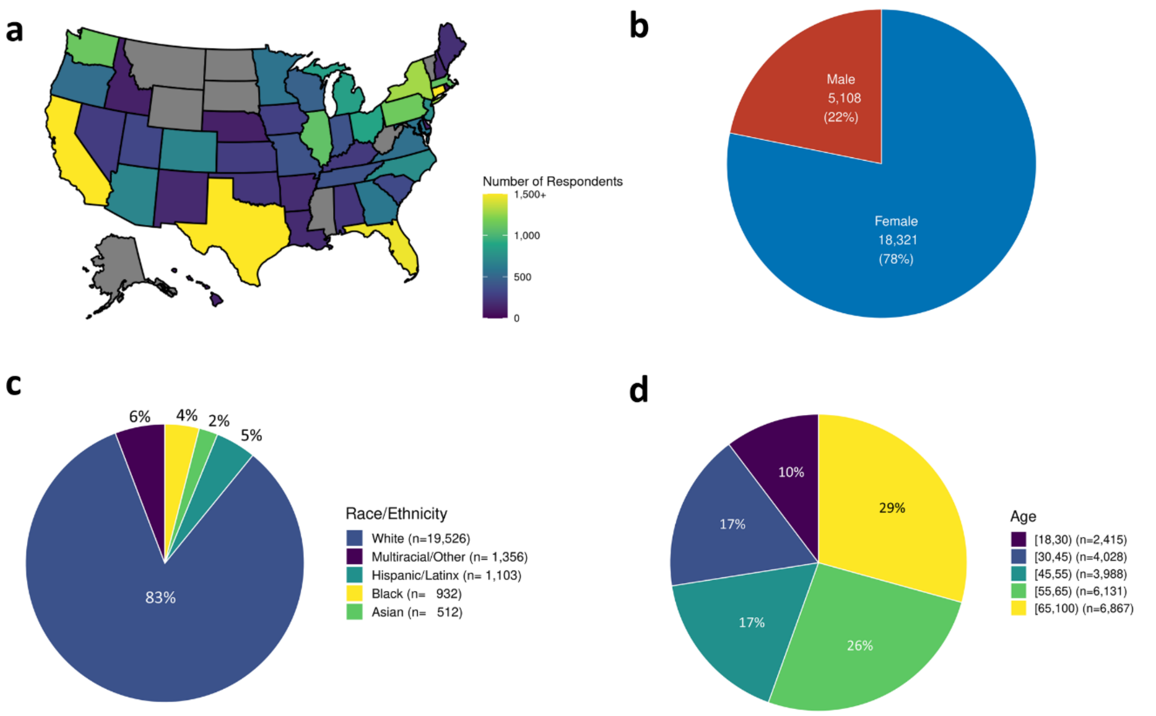

Supplementary Figure 3, HWF Vaccine Uptake Demographic Distributions : Demographic break down of the HWF user base that responded to the vaccine uptake question by **(a)** state, **(b)** sex, **(c)** race and **(d)** age.

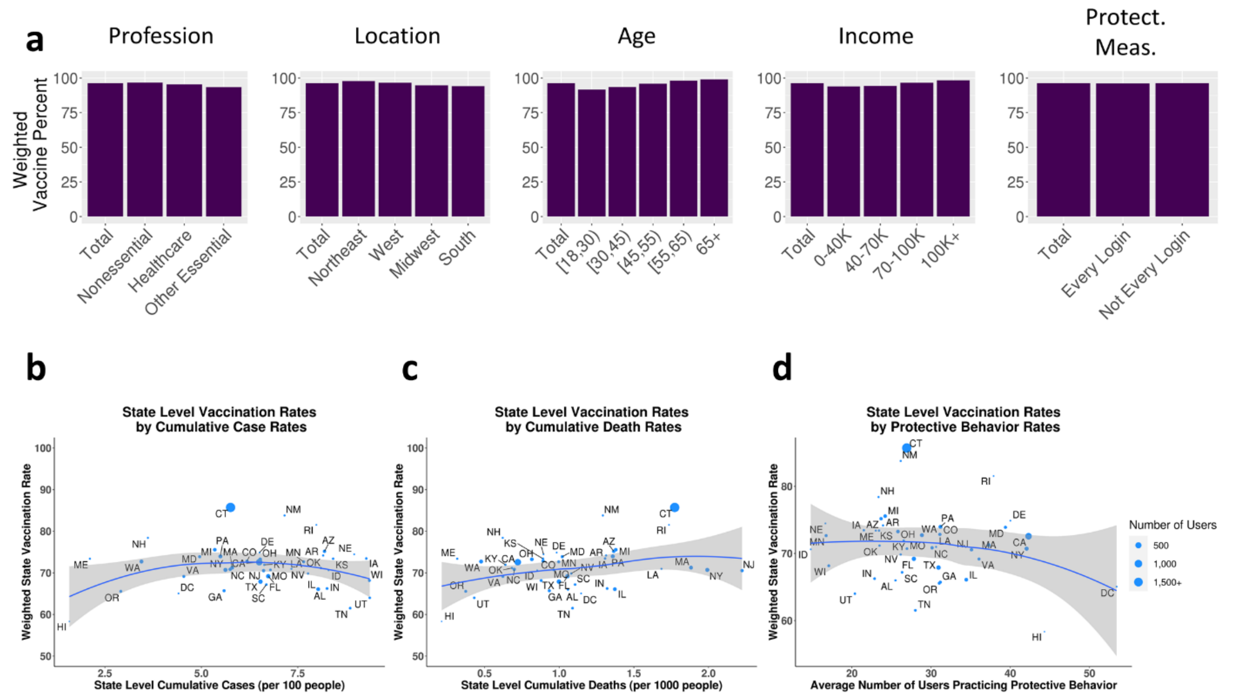

Supplementary Figure 4, Demographic specific weighted vaccine uptake rates: (a) Weighted vaccination rates of users that were offered a vaccine by profession, location, age, income, and use of protective measures. State level weighted vaccination rates of users that were offered a vaccine by **(c)** cumulative case rates (/100 individuals), **(d)** cumulative death rates (/1000 individuals), **(e)** and average number of users practicing protective behavior.

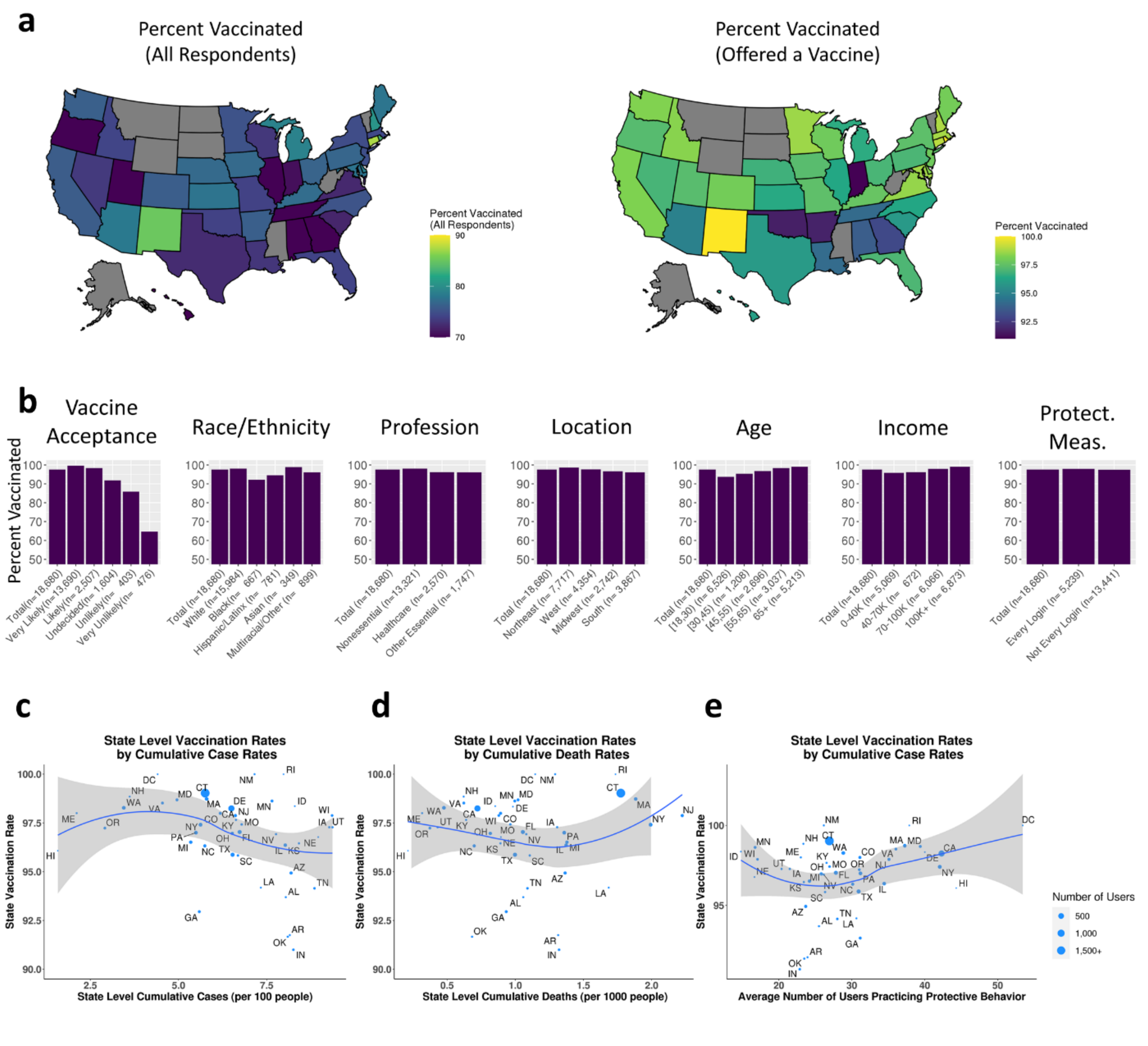

Supplementary Figure 5, Demographic Specific Vaccine Uptake Rates: (a) Unweighted vaccination rates by state of (Left) all users that responded to the vaccine uptake question and (right) users that were offered a vaccine. **(b)** Vaccination rates of users that were offered a vaccine by vaccine acceptance, race/ethnicity, profession, location, age, income, and use of protective measures. **(c)** State level vaccination rates of users that were offered a vaccine by **(b)** cumulative case rates (/100 individuals), **(c)** cumulative death rates (/1000 individuals), **(d)** and average number of users practicing protective behavior.

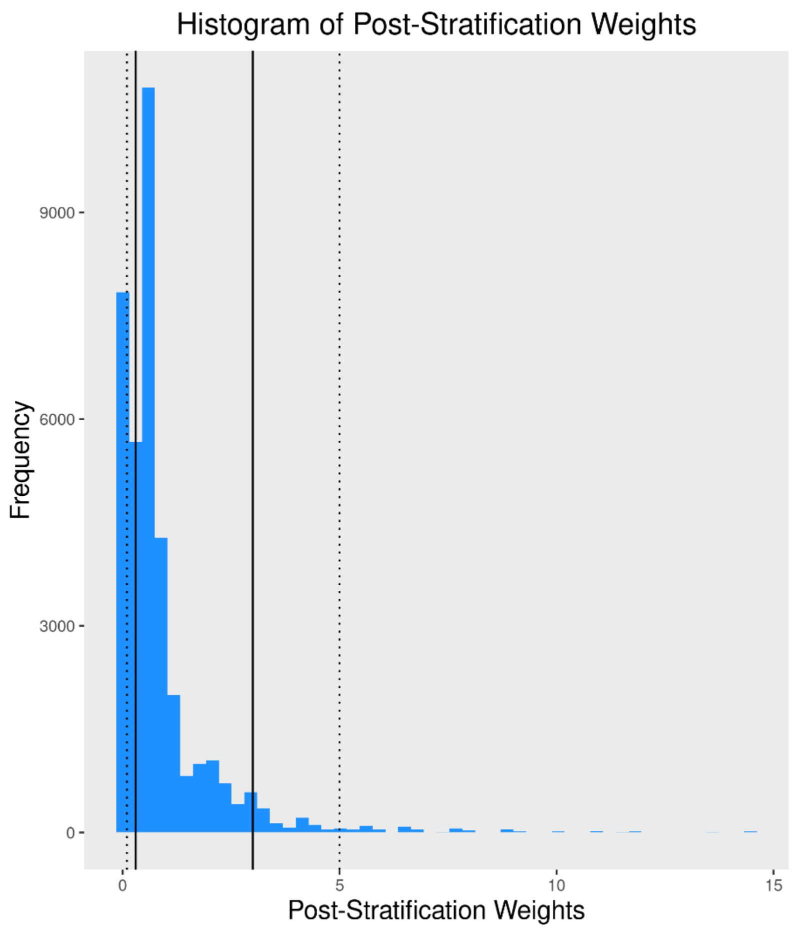

Supplementary Figure 6, Post-Stratification Weights: Histogram of post-stratification weights adjusted for census location, race, age, and sex. Solid lines indicate the trimming threshold and the dotted line indicates the trimming threshold for the sensitivity analysis.

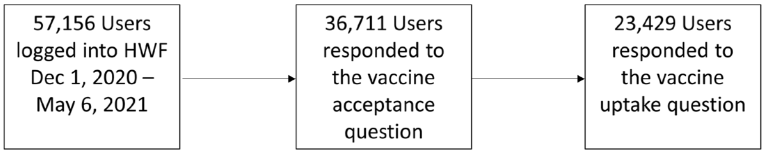

Supplementary Figure 7, Consort Diagram: Diagram of number of individuals answering each question in the How We Feel Application.
