# Supplementary material for: Unraveling attributes of COVID-19 vaccine acceptance and uptake in the U.S.: a large nationwide study
Source: Sci Rep. 2023 May 24;13:8360. doi: 10.1038/s41598-023-34340-3 (PMC10209066; doi:10.1038/s41598-023-34340-3)
Supplement: Supplementary file 2 — Supplementary Information 2. [file 41598_2023_34340_MOESM2_ESM.pdf]

Supplementary Table 1: Demographics – Descriptive statistics for the demographic characteristics of the HWF user base.

Variable	Level	Acceptance Respondents (n = 36,711)	Uptake Respondents (n=23,429)
**Sex**			
	**Male**	7,616 (21%)	5,108 (22%)
	**Female**	29,095 (79%)	18,321 (78%)
**Age**			
	**[18,30)**	4,815 (13%)	2,415 (10%)
	**[30,45)**	7,468 (20%)	4,028 (17%)
	**[45,55)**	6,416 (17%)	3,988 (17%)
	**[55,65)**	8,767 (24%)	6,131 (26%)
	**[65,100)**	9,245 (25%)	6,867 (29%)
**Profession**			
	**Nonessential**	24,823 (68%)	16,492 (70%)
	**Healthcare**	4,597 (13%)	2,773 (12%)
	**Other Essential**	4,087 (11%)	2,529 (11%)
	**Missing**	3,204 (9%)	1,635 (7%)
**Employment Status**			
	**Employed**	19,205 (52%)	12,206 (52%)
	**Furloughed/Job Seeker**	2,344 (6%)	1,466 (6%)
	**Unemployed**	10,264 (28%)	7,581 (32%)
	**Missing**	4,898 (13%)	2,176 (9%)
**Race/Ethnicity**			
	**White**	30,128 (83%)	19,526 (83%)
	**Black/African American**	1,461 (4%)	932 (4%)
	**Hispanic/Latinx**	1,968 (5%)	1,103 (5%)
	**Asian**	904 (2%)	512 (2%)
	**Multiracial/Other**	2,250 (6%)	1,356 (6%)
**Median Household Income**			
	**0-40k**	1,458 (4%)	897 (4%)
	**40-70k**	12,478 (34%)	7,770 (33%)
	**70-100k**	13,151 (36%)	8,536 (36%)
	**100K+**	9,624 (26%)	6,226 (27%)
**Population Density**			
	**0-149 people/sq. mi**	4,079 (11%)	2,509 (11%)
	**150-999 people/sq. mi**	13,516 (37%)	8,497 (36%)
	**1000+ people/sq. mi**	19,116 (52%)	12,423 (53%)
**Preexisting Conditions**			
	**0**	8,535 (23%)	5,266 (22%)
	**1**	12,564 (34%)	7,905 (34%)
	**2**	8,502 (23%)	5,446 (23%)
	**3+**	6,723 (18%)	4,589 (20%)
	**Not Say**	387 (1%)	223 (1%)
**Census Location**			
	**Northeast**	13,790 (38%)	9,139 (39%)
	**West**	9,053 (25%)	5,649 (24%)
	**Midwest**	5,627 (15%)	3,561 (15%)
	**South**	8,241 (22%)	5,080 (22%)
**Practiced Protective Measures**			
	**During Every Login**	13,108 (36%)	7,146 (31%)
	**Not Every Login**	23,603 (64%)	16,283 (69%)
**Ever Received COVID Test**			
	**Yes**	5,075 (14%)	3,262 (14%)
	**No**	31,636 (86%)	20,167 (86%)
**COVID Status**			
	**Never Tested Positive/Never Tested**	36,536 (99%)	107 (<1%)
	**Tested Positive**	175 (<1%)	23,322 (99%)

Supplementary Table 2: Hesitancy Rates – Unweighted and weighted percentages of the vaccine acceptance responses across demographics.

		A Unweighted Percents					B Weighted Percents				
**Variable**	**Level**	**Very Unlikely (4%)**	**Unlikely (3%)**	**Undecided (10%)**	**Likely (13%)**	**Very Likely (70%)**	**Very Unlikely (4%)**	**Unlikely (3%)**	**Undecide d (11%)**	**Likely (13%)**	**Very Likely (69%)**
**Sex**											
	**Male**	188 (2%)	141 (2%)	487 (6%)	765 (10%)	6035 (79%)	3%	2%	8%	11%	76%
	**Female**	1140 (4%)	802 (3%)	3335 (11%)	3980 (14%)	19838 (68%)	5%	3%	14%	14%	64%
**Age**											
	**[18,30)**	237 (5%)	152 (3%)	620 (13%)	747 (16%)	3059 (64%)	6%	3%	14%	15%	62%
	**[30,45)**	352 (5%)	227 (3%)	947 (13%)	1028 (14%)	4914 (66%)	5%	3%	13%	13%	65%
	**[45,55)**	296 (5%)	175 (3%)	821 (13%)	866 (13%)	4258 (66%)	5%	3%	13%	13%	66%
	**[55,65)**	236 (3%)	196 (2%)	810 (9%)	1065 (12%)	6460 (74%)	3%	2%	10%	12%	73%
	**[65,100)**	207 (2%)	193 (2%)	624 (7%)	1039 (11%)	7182 (78%)	3%	2%	7%	11%	77%
**Profession**											
	**Nonessential**	793 (3%)	603 (2%)	2345 (9%)	3241 (13%)	17841 (72%)	4%	3%	10%	13%	70%
	**Healthcare**	177 (4%)	130 (3%)	551 (12%)	548 (12%)	3191 (69%)	4%	3%	13%	12%	68%
	**Other Essential**	217 (5%)	139 (3%)	581 (14%)	580 (14%)	2570 (63%)	6%	4%	14%	14%	62%
	**Missing**	141 (4%)	71 (2%)	345 (11%)	376 (12%)	2271 (71%)	5%	2%	12%	12%	69%
**Self-Reported Race/Ethnicity**											
	**White**	904 (3%)	682 (2%)	2696 (9%)	3755 (12%)	22091 (73%)	3%	2%	8%	12%	74%
	**Black/African American**	155 (11%)	96 (7%)	358 (25%)	235 (16%)	617 (42%)	11%	7%	25%	16%	42%
	**Hispanic/Latinx**	120 (6%)	70 (4%)	346 (18%)	258 (13%)	1174 (60%)	6%	3%	17%	13%	60%
	**Asian**	18 (2%)	22 (2%)	119 (13%)	151 (17%)	594 (66%)	2%	3%	14%	15%	66%
	**Multiracial/Other**	131 (6%)	73 (3%)	303 (13%)	346 (15%)	1397 (62%)	6%	3%	13%	14%	65%
**Median Household Income**											

	**0-40k**	85 (6%)	62 (4%)	234 (16%)	170 (12%)	907 (62%)	6%	5%	17%	11%	61%
	**40-70k**	618 (5%)	393 (3%)	1605 (13%)	1645 (13%)	8217 (66%)	6%	3%	14%	13%	65%
	**70-100k**	402 (3%)	312 (2%)	1281 (10%)	1715 (13%)	9441 (72%)	4%	2%	10%	13%	71%
	**100K+**	223 (2%)	176 (2%)	702 (7%)	1215 (13%)	7308 (76%)	3%	2%	8%	13%	75%
**Population Density**											
	**0-149 people/sq. mi**	199 (5%)	131 (3%)	542 (13%)	575 (14%)	2632 (65%)	5%	3%	14%	14%	65%
	**150-999 people/sq. mi**	579 (4%)	379 (3%)	1448 (11%)	1726 (13%)	9384 (69%)	5%	3%	11%	12%	68%
	**1000+ people/sq. mi**	550 (3%)	433 (2%)	1832 (10%)	2444 (13%)	13857 (72%)	4%	3%	11%	13%	70%
**Preexisting Conditions**											
	**0**	295 (3%)	197 (2%)	869 (10%)	1121 (13%)	6053 (71%)	4%	3%	11%	13%	69%
	**1**	412 (3%)	343 (3%)	1336 (11%)	1618 (13%)	8855 (70%)	4%	3%	12%	13%	68%
	**2**	302 (4%)	231 (3%)	840 (10%)	1119 (13%)	6010 (71%)	4%	3%	11%	12%	70%
	**3+**	293 (4%)	164 (2%)	734 (11%)	838 (12%)	4694 (70%)	5%	3%	12%	12%	69%
	**Not Say**	26 (7%)	8 (2%)	43 (11%)	49 (13%)	261 (67%)	9%	2%	14%	14%	61%
**Census Location**											
	**Northeast**	345 (3%)	277 (2%)	1255 (9%)	1780 (13%)	10133 (73%)	3%	2%	10%	13%	71%
	**West**	307 (3%)	240 (3%)	902 (10%)	1174 (13%)	6430 (71%)	3%	3%	10%	13%	71%
	**Midwest**	216 (4%)	164 (3%)	641 (11%)	715 (13%)	3891 (69%)	4%	3%	11%	13%	70%
	**South**	460 (6%)	262 (3%)	1024 (12%)	1076 (13%)	5419 (66%)	6%	3%	13%	13%	65%
**Employment Status**											
	**Employed**	661 (3%)	512 (3%)	2108 (11%)	2619 (14%)	13305 (69%)	4%	3%	11%	14%	69%
	**Furloughed/Job Seeker**	102 (4%)	90 (4%)	303 (13%)	310 (13%)	1539 (66%)	5%	4%	16%	12%	63%
	**Unemployed**	320 (3%)	227 (2%)	808 (8%)	1287 (13%)	7622 (74%)	4%	2%	9%	12%	72%
	**Missing**	245 (5%)	114 (2%)	603 (12%)	529 (11%)	3407 (70%)	6%	3%	13%	11%	67%
**Practiced Protective Measures**											
	**During Every Login**	438 (3%)	287 (2%)	1282 (10%)	1475 (11%)	9626 (73%)	4%	2%	11%	11%	72%

	**Not Every Login**	890 (4%)	656 (3%)	2540 (11%)	3270 (14%)	16247 (69%)	4%	3%	12%	14%	67%
**Ever Received COVID Test**											
	**Yes**	145 (3%)	100 (2%)	462 (9%)	569 (11%)	3799 (75%)	3%	2%	10%	11%	74%
	**No**	1183 (4%)	843 (3%)	3360 (11%)	4176 (13%)	22074 (70%)	4%	3%	12%	13%	68%

Supplementary Table 3: Univariate Analysis – Unweighted and weighted univariate logistic regression analyses for vaccine reluctance.

Variable	Reference Group	A Univariate Analysis					B Weighted Univariate Analysis				
		OR	Lower 95% CI	Upper 95% CI	P-Value	Sig	OR	Lower 95% CI	Upper 95% CI	P-Value	Sig
Female	Male	1.85	1.71	2.00	6.49E-53	***	1.91	1.74	2.09	2.54E-43	***
Age [18,30)	65+	2.13	1.94	2.34	1.07E-54	***	2.10	1.87	2.36	4.58E-35	***
Age [30,45)		2.06	1.89	2.25	3.33E-61	***	1.93	1.73	2.15	4.81E-32	***
Age [45,55)		2.02	1.85	2.21	2.94E-54	***	1.90	1.69	2.13	1.13E-27	***
Age [55,65)		1.33	1.21	1.45	4.44E-10	***	1.24	1.11	1.39	0.0002	**
Essential Healthcare	Nonessential	1.29	1.19	1.40	7.76E-10	***	1.32	1.19	1.46	1.01E-07	***
Other Essential		1.68	1.55	1.82	5.03E-36	***	1.56	1.41	1.73	2.25E-17	***
Furloughed/Job Seeker	Employed	1.30	1.17	1.44	1.31E-06	***	1.52	1.33	1.73	6.56E-10	***
Unemployed		0.74	0.69	0.79	3.34E-18	***	0.83	0.76	0.90	2.39E-05	***
African American/Black	White	4.31	3.87	4.81	1.52E-152	***	4.52	4.01	5.10	1.14E-131	***
Hispanic/Latinx		2.26	2.03	2.51	7.17E-53	***	2.24	2.00	2.52	2.73E-43	***
Asian		1.29	1.08	1.53	0.0044	*	1.46	1.21	1.77	0.0001	**
Multiracial/Other		1.76	1.58	1.95	2.89E-26	***	1.66	1.47	1.89	2.37E-15	***
1 Preexisting	0	1.05	0.98	1.13	0.1794		1.05	0.95	1.15	0.3305	
2 Preexisting		1.02	0.94	1.10	0.7181		1.01	0.91	1.12	0.8746	
3+ Preexisting		1.13	1.04	1.24	0.0037	*	1.08	0.97	1.21	0.1479	
Preexisting Not Say		1.31	1.01	1.68	0.0392	*	1.51	1.08	2.09	0.0146	*
Parent	Not a Parent	1.04	0.69	1.52	0.8458		1.17	0.72	1.90	0.5339	
Income 0-40K	100K+	0.96	0.91	1.02	0.190		0.99	0.92	1.06	0.7220	
Income 40-70K		2.74	2.40	3.12	3.95E-50	***	2.69	2.29	3.16	1.50E-33	***
Income 70-100K		2.05	1.90	2.22	1.41E-76	***	2.03	1.84	2.24	8.79E-46	***
PopDensity 0-149	1000+	1.38	1.28	1.50	6.02E-16	***	1.36	1.22	1.50	6.30E-09	***
PopDensity 150-999		1.57	1.45	1.71	1.03E-25	***	1.37	1.24	1.53	2.11E-09	***
West	Northeast	1.25	1.18	1.33	9.01E-14	***	1.22	1.13	1.32	2.20E-07	***
Midwest		1.21	1.12	1.30	5.33E-07	***	1.07	0.97	1.18	0.1640	
South		1.41	1.29	1.53	1.08E-15	***	1.18	1.06	1.31	0.0026	*

**Practiced Protective Behavior**	**Did Not Practice**	1.06	1.05	1.07	1.32E-38	***	1.04	1.03	1.05	8.19E-15	***
**Answered before Pfizer EUA**	**After Pfizer EUA**	1.15	1.09	1.21	8.79E-07	***	1.15	1.07	1.23	0.0001	**
**State Cumulative Case Rate**		1.71	1.59	1.83	3.99E-48	***	1.52	1.39	1.67	4.50E-19	***
**State Cumulative Death Rate**		1.09	1.07	1.11	5.12E-16	***	1.05	1.02	1.08	8.63E-05	***
**Average Daily Pos Test Rate (Nov.)**		0.85	0.81	0.90	1.64E-08	***	0.96	0.90	1.04	0.3131	
**County % without HS Degree**		1.03	1.03	1.04	3.11E-30	***	1.03	1.02	1.04	2.45E-16	***
**Received COVID Test**	**Never Received Test**	0.79	0.72	0.86	4.05E-08	***	0.78	0.70	0.87	9.41E-06	***

*: p < 0.05
**: p < 0.001
***: p < 0.0001

Supplementary Table 4: Multivariable Analysis –
Unweighted and weighted multivariable logistic regression analyses for vaccine reluctance.

		A					B				
		Multivariable Unweighted Analysis					Multivariable Weighted Analysis				
**Variable**	**Reference Group**	**OR**	**Lower 95% CI**	**Upper 95% CI**	**P-Value**	**Sig**	**OR**	**Lower 95% CI**	**Upper 95% CI**	**P-Value**	**Sig**
**Female**	**Male**	1.73	1.59	1.88	1.41E-38	***	1.67	1.51	1.83	4.09E-25	***
**Age [18,30)**	**65+**	1.96	1.73	2.21	3.27E-27	***	2.17	1.86	2.53	1.03E-22	***
**Age [30,45)**		1.94	1.75	2.15	1.15E-37	***	2.02	1.77	2.29	8.92E-27	***
**Age [45,55)**		1.89	1.71	2.10	9.39E-35	***	1.96	1.72	2.23	1.52E-23	***
**Age [55,65)**		1.26	1.15	1.39	2.11E-06	***	1.27	1.12	1.44	0.0002	**
**Essential Healthcare**	**Nonessential**	1.18	1.08	1.29	0.0002	**	1.20	1.08	1.33	0.0010	*
**Other Essential**		1.57	1.44	1.72	1.91E-24	***	1.52	1.36	1.70	1.42E-13	***
**Furloughed/Job Seeker**	**Employed**	1.30	1.16	1.45	4.40E-06	***	1.48	1.29	1.70	4.04E-08	***
**Unemployed**		0.99	0.91	1.08	0.8267		1.13	1.01	1.25	0.0275	*
**African American/Black**	**White**	3.84	3.42	4.31	2.80E-114	***	3.94	3.47	4.48	1.26E-96	***
**Hispanic/Latinx**		1.85	1.65	2.07	1.59E-26	***	1.89	1.67	2.14	1.99E-23	***
**Asian**		1.49	1.24	1.78	1.71E-05	***	1.80	1.46	2.21	2.05E-08	***
**Multiracial/Other**		1.55	1.39	1.73	3.10E-15	***	1.59	1.39	1.81	8.11E-12	***
**1 Preexisting**	**0**	1.07	0.99	1.15	0.0954		1.06	0.96	1.17	0.2144	
**2 Preexisting**		1.04	0.95	1.13	0.4081		1.02	0.92	1.14	0.6578	
**3+ Preexisting**		1.25	1.14	1.37	2.47E-06	***	1.19	1.06	1.34	0.0036	*
**Preexisting Not Say**		1.39	1.05	1.81	0.0169	*	1.58	1.09	2.30	0.0161	*
**Parent**	**Not a Parent**	1.20	1.12	1.29	2.25E-07	***	1.26	1.15	1.38	9.61E-07	***
**Income 0-40K**	**100K+**	1.64	1.42	1.90	4.20E-11	***	1.66	1.39	1.98	2.33E-08	***
**Income 40-70K**		1.56	1.43	1.70	2.69E-24	***	1.58	1.42	1.77	1.85E-16	***
**Income 70-100K**		1.24	1.14	1.35	2.85E-07	***	1.21	1.08	1.34	0.0005	**
**PopDensity 0-149**	**1000+**	1.34	1.21	1.47	4.84E-09	***	1.32	1.18	1.49	3.86E-06	***
**PopDensity 150-999**		1.22	1.14	1.30	5.18E-09	***	1.27	1.16	1.38	5.02E-08	***
**West**	**Northeast**	1.01	0.87	1.18	0.8896		1.02	0.83	1.23	0.8805	
**Midwest**		1.12	0.97	1.28	0.1227		1.11	0.93	1.32	0.2364	
**South**		1.28	1.12	1.47	0.0004	**	1.25	1.05	1.48	0.0105	*
**Answered before Pfizer EUA**	**After Pfizer EUA**	1.46	1.37	1.55	5.32E-33	***	1.48	1.37	1.60	9.96E-23	***

**Practiced Protective Behavior**	**Did Not Practice**	0.79	0.74	0.84	6.95E-13 ***	0.78	0.72	0.85	6.12E-09 ***
**State Cumulative Case Rate**		1.00	0.97	1.03	0.9449	1.01	0.97	1.04	0.7826
**State Cumulative Death Rate**		1.20	1.07	1.36	0.0030 *	1.18	1.01	1.38	0.0351 *
**Average Daily Pos Test Rate (Nov.)**		1.01	0.99	1.02	0.4374	1.00	0.99	1.02	0.9654
**County % without HS Degree**		1.02	1.01	1.02	3.21E-05 ***	1.01	1.00	1.02	0.0819
**Received COVID Test**	**Never Received Test**	0.79	0.72	0.87	3.10E-07 ***	0.79	0.71	0.89	5.88E-05 ***

*: $p < 0.05$
**: $p < 0.001$
***: $p < 0.0001$

Supplementary Table 5: Nominal Unweighted – Unweighted nominal logistic regression analyses comparing "Undecided" vs "Likely" (Left) and "Unlikely" vs "Likely" for vaccine acceptance.

		A Undecided					B Unlikely				
**Variable**	**Reference Group**	**OR**	**Lower 95% CI**	**Upper 95% CI**	**P-Value**	**Sig**	**OR**	**Lower 95% CI**	**Upper 95% CI**	**P-Value**	**Sig**
**Female**	**Male**	1.88	1.70	2.08	2.68E-33	***	1.51	1.33	1.71	1.18E-10	***
**Age [18,30)**	**65+**	1.91	1.65	2.22	1.29E-17	***	2.04	1.69	2.45	4.23E-14	***
**Age [30,45)**		1.92	1.69	2.17	7.23E-25	***	1.98	1.70	2.31	3.37E-18	***
**Age [45,55)**		1.92	1.70	2.18	4.64E-25	***	1.84	1.58	2.15	1.39E-14	***
**Age [55,65)**		1.31	1.17	1.48	4.64E-06	***	1.17	1.01	1.36	0.0387	*
**Essential Healthcare**	**Nonessential**	1.18	1.06	1.31	0.0022	*	1.18	1.03	1.35	0.0152	*
**Other Essential**		1.56	1.41	1.73	7.61E-17	***	1.60	1.40	1.82	1.42E-12	***
**Furloughed/Job Seeker**	**Employed**	1.25	1.09	1.43	0.0013	*	1.39	1.18	1.64	9.21E-05	***
**Unemployed**		0.92	0.83	1.02	0.1067		1.12	0.99	1.27	0.0769	*
**African American/Black**	**White**	3.63	3.17	4.17	1.33E-75	***	4.19	3.57	4.91	5.62E-70	***
**Hispanic/Latinx**		1.95	1.71	2.23	1.25E-22	***	1.69	1.43	2.01	1.96E-09	***
**Asian**		1.77	1.44	2.18	5.61E-08	***	1.01	0.73	1.41	0.9298	
**Multiracial/Other**		1.51	1.32	1.72	2.02E-09	***	1.63	1.39	1.91	2.33E-09	***
**1 Preexisting**	**0**	1.07	0.97	1.17	0.1557		1.07	0.95	1.20	0.3002	
**2 Preexisting**		1.00	0.90	1.11	0.9525		1.11	0.97	1.26	0.1259	
**3+ Preexisting**		1.21	1.08	1.35	0.0007	**	1.31	1.14	1.51	0.0001	**
**Preexisting Not Say**		1.21	0.86	1.70	0.2642		1.71	1.17	2.49	0.0058	*
**Parent**	**Not a Parent**	1.17	1.07	1.27	0.0003	**	1.27	1.14	1.41	1.77E-05	***
**Income 0-40K**	**100K+**	1.69	1.42	2.02	5.78E-09	***	1.56	1.26	1.94	6.17E-05	***
**Income 40-70K**		1.57	1.42	1.75	1.86E-17	***	1.54	1.35	1.76	1.81E-10	***
**Income 70-100K**		1.28	1.16	1.41	1.56E-06	***	1.18	1.04	1.34	0.0126	*
**PopDensity 0-149**	**1000+**	1.31	1.17	1.48	5.08E-06	***	1.38	1.19	1.60	1.61E-05	***
**PopDensity 150-999**		1.13	1.05	1.23	0.0022	*	1.38	1.24	1.52	6.71E-10	***
**West**	**Northeast**	0.95	0.79	1.14	0.5796		1.14	0.90	1.44	0.2846	
**Midwest**		1.03	0.87	1.22	0.7064		1.28	1.04	1.59	0.0214	*
**South**		1.13	0.96	1.34	0.1353		1.58	1.28	1.94	1.58E-05	***
**Answered before Pfizer EUA**	**After Pfizer EUA**	1.59	1.47	1.71	5.40E-34	***	1.26	1.15	1.38	1.52E-06	***

**Practiced Protective Behavior**	**Did Not Practice**	0.82	0.76	0.89	8.19E-07	***	0.73	0.66	0.81	1.59E-09	***
**State Cumulative Case Rate**		1.01	0.97	1.05	0.7091		0.99	0.95	1.04	0.7155	
**State Cumulative Death Rate**		1.21	1.04	1.41	0.0116	*	1.19	0.99	1.44	0.0638	
**Average Daily Pos Test Rate (Nov.)**		1.01	0.99	1.03	0.1945		1.00	0.98	1.02	0.6997	
**County % without HS Degree**		1.01	1.00	1.02	0.0152	*	1.02	1.01	1.03	3.29E-05	***
**Received COVID Test**	**Never Received Test**	0.82	0.74	0.92	0.0004	**	0.74	0.64	0.85	2.44E-05	***

*: $p < 0.05$
**: $p < 0.001$
***: $p < 0.0001$

Supplementary Table 6: Nominal Weighted –
Weighted nominal logistic regression analyses for vaccine acceptance.

		A					B				
		Undecided Weighted					Unlikely Weighted				
Variable	Reference Group	OR	Lower 95% CI	Upper 95% CI	P-Value	Sig	OR	Lower 95% CI	Upper 95% CI	P-Value	Sig
**Female**	**Male**	1.80	1.59	2.02	1.21E-21	***	1.51	1.29	1.72	1.05E-07	***
**Age [18,30)**	**65+**	1.94	1.61	2.35	6.09E-12	***	2.04	2.01	3.24	1.40E-14	***
**Age [30,45)**		1.94	1.65	2.27	3.08E-16	***	1.98	1.78	2.63	7.86E-15	***
**Age [45,55)**		1.98	1.68	2.32	2.23E-16	***	1.84	1.58	2.36	1.43E-10	***
**Age [55,65)**		1.31	1.13	1.53	0.0005	**	1.17	0.97	1.43	0.0954	
**Essential Healthcare**	**Nonessential**	1.22	1.07	1.39	0.0028	*	1.18	0.98	1.37	0.0813	
**Other Essential**		1.49	1.30	1.71	7.14E-09	***	1.60	1.32	1.83	1.70E-07	***
**Furloughed/Job Seeker**	**Employed**	1.50	1.27	1.78	2.61E-06	***	1.39	1.18	1.78	0.0004	**
**Unemployed**		1.07	0.94	1.22	0.3332		1.12	1.04	1.44	0.0133	*
**African American/Black**	**White**	3.90	3.34	4.56	1.39E-66	***	4.19	3.38	4.79	1.65E-54	***
**Hispanic/Latinx**		2.04	1.76	2.37	6.98E-21	***	1.69	1.39	2.03	8.68E-08	***
**Asian**		2.12	1.68	2.68	2.56E-10	***	1.01	0.86	1.76	0.2606	
**Multiracial/Other**		1.56	1.32	1.83	7.17E-08	***	1.63	1.35	1.99	8.13E-07	***
**1 Preexisting**	**0**	1.08	0.96	1.22	0.1770		1.07	0.89	1.21	0.6269	
**2 Preexisting**		0.96	0.85	1.10	0.5888		1.11	0.95	1.32	0.1822	
**3+ Preexisting**		1.15	1.00	1.33	0.0535		1.31	1.02	1.46	0.0266	*
**Preexisting Not Say**		1.33	0.83	2.13	0.2373		1.71	1.12	3.12	0.0169	*
**Parent**	**Not a Parent**	1.17	1.05	1.31	0.0052	*	1.27	1.26	1.68	2.86E-07	***
**Income 0-40K**	**100K+**	1.75	1.41	2.18	3.26E-07	***	1.56	1.21	2.05	0.0008	**
**Income 40-70K**		1.62	1.42	1.85	1.74E-12	***	1.54	1.31	1.83	3.75E-07	***
**Income 70-100K**		1.25	1.10	1.42	0.0008	**	1.18	0.97	1.35	0.1152	
**PopDensity 0-149**	**1000+**	1.31	1.14	1.51	0.0002	**	1.38	1.11	1.59	0.0019	**
**PopDensity 150-999**		1.17	1.06	1.30	0.0025	*	1.38	1.24	1.59	1.52E-07	***
**West**	**Northeast**	0.96	0.75	1.22	0.7255		1.14	0.83	1.49	0.4735	
**Midwest**		1.03	0.84	1.28	0.7637		1.28	0.97	1.62	0.0827	
**South**		1.10	0.89	1.36	0.3716		1.58	1.19	1.97	0.0008	**
**Answered before Pfizer EUA**	**After Pfizer EUA**	1.60	1.45	1.76	1.51E-21	***	1.26	1.18	1.50	2.68E-06	***
**Mask Wearing/Protective Measures**	**Did Not Practice**	0.82	0.74	0.90	8.80E-05	***	0.73	0.64	0.83	1.56E-06	***

**State Cumulative Case Rate**		1.01	0.97	1.06	0.5969		0.99	0.94	1.05	0.8213	
**State Cumulative Death Rate**		1.18	0.97	1.43	0.0919		1.19	0.94	1.49	0.1448	
**Average Daily Pos Test Rate (Nov.)**		1.00	0.99	1.02	0.6512		1.00	0.97	1.02	0.5181	
**County % without HS Degree**		1.01	1.00	1.02	0.2902		1.02	1.00	1.02	0.1312	
**Received COVID Test**	**Never Received Test**	0.82	0.72	0.94	0.0050	*	0.74	0.62	0.89	0.0013	*

*: $p < 0.05$
**: $p < 0.001$
***: $p < 0.0001$

Supplementary Table 7: Sensitivity Multivariable – Sensitivity analysis for the multivariable logistic regression analysis for vaccine reluctance using a new weight threshold of 0.1 to 5.

		Multivariable Sensitivity Analysis for Weight Thresholds (0.1,5)				
**Variable**	**Reference Group**	**OR**	**Lower 95% CI**	**Upper 95% CI**	**P-Value**	**Significant**
**Female**	**Male**	1.65	1.48	1.83	5.93E-21	***
**Age [18,30)**	**65+**	2.26	1.91	2.68	6.15E-21	***
**Age [30,45)**		2.03	1.77	2.34	4.37E-23	***
**Age [45,55)**		1.98	1.71	2.28	1.05E-20	***
**Age [55,65)**		1.27	1.11	1.46	0.0005	**
**Essential Healthcare**	**Nonessential**	1.20	1.07	1.35	0.0022	*
**Other Essential**		1.50	1.33	1.70	1.35E-10	***
**Furloughed/Job Seeker**	**Employed**	1.48	1.27	1.72	4.37E-07	***
**Unemployed**		1.17	1.04	1.31	0.0097	*
**African American/Black**	**White**	3.83	3.33	4.39	4.11E-80	***
**Hispanic/Latinx**		1.91	1.67	2.19	7.94E-21	***
**Asian**		1.80	1.46	2.22	3.93E-08	***
**Multiracial/Other**		1.57	1.36	1.81	1.24E-09	***
**1 Preexisting**	**0**	1.07	0.96	1.19	0.2529	
**2 Preexisting**		1.02	0.90	1.15	0.7673	
**3+ Preexisting**		1.19	1.05	1.36	0.0082	*
**Preexisting Not Say**		1.61	1.06	2.43	0.0248	*
**Parent**	**Not a Parent**	1.28	1.16	1.42	2.28E-06	***
**Income 0-40K**	**100K+**	1.70	1.40	2.08	1.40E-07	***
**Income 40-70K**		1.59	1.41	1.80	1.02E-13	***
**Income 70-100K**		1.19	1.06	1.34	0.0040	*
**PopDensity 0-149**	**1000+**	1.30	1.14	1.48	9.26E-05	***
**PopDensity 150-999**		1.29	1.17	1.42	1.90E-07	***
**West**	**Northeast**	1.01	0.81	1.25	0.9410	
**Midwest**		1.10	0.90	1.33	0.3495	
**South**		1.26	1.04	1.52	0.0180	*
**Answered before Pfizer EUA**	**After Pfizer EUA**	1.47	1.35	1.61	2.73E-18	***
**Practiced Protective Behavior**	**Did Not Practice**	0.78	0.71	0.85	1.04E-07	***
**State Cumulative Case Rate**		1.01	0.97	1.05	0.6396	

**State Cumulative Death Rate**		1.17	0.99	1.39	0.0650	
**Average Daily Pos Test Rate (Nov.)**		1.00	0.98	1.01	0.7254	
**County % without HS Degree**		1.00	1.00	1.01	0.2847	
**Received COVID Test**	**Never Received Test**	0.78	0.68	0.88	6.52E-05	***

*: p < 0.05
**: p < 0.001
***: p < 0.0001

Supplementary Table 8: Sensitivity Nominal - Sensitivity analysis for the nominal logistic regression analysis for vaccine acceptance using a new weight threshold of 0.1 to 5. Left: logistic regression analyses comparing “Undecided” vs “Likely” and right: logistic regression analyses comparing “Unlikely” vs “Likely”.

		A Undecided Sensitivity Weights (0.1, 5)					B Unlikely Sensitivity Weights (0.1, 5)				
**Variable**	**Reference Group**	**OR**	**Lower 95% CI**	**Upper 95% CI**	**P-Value**	**Sig**	**OR**	**Lower 95% CI**	**Upper 95% CI**	**P-Value**	**Sig**
**Female**	**Male**	1.79	1.57	2.03	1.08E-18	***	1.46	1.25	1.71	2.57E-06	***
**Age [18,30)**	**65+**	1.92	1.56	2.36	5.62E-10	***	2.87	2.21	3.73	3.53E-15	***
**Age [30,45)**		1.91	1.61	2.28	1.79E-13	***	2.24	1.82	2.77	8.28E-14	***
**Age [45,55)**		1.97	1.65	2.35	4.60E-14	***	1.98	1.59	2.46	5.94E-10	***
**Age [55,65)**		1.30	1.10	1.54	0.0019	*	1.20	0.98	1.48	0.0848	
**Essential Healthcare**	**Nonessential**	1.23	1.06	1.41	0.0049	*	1.16	0.97	1.39	0.1058	
**Other Essential**		1.48	1.27	1.72	3.81E-07	***	1.52	1.27	1.82	6.39E-06	***
**Furloughed/Job Seeker**	**Employed**	1.52	1.27	1.83	6.91E-06	***	1.42	1.13	1.78	0.0022	*
**Unemployed**		1.10	0.96	1.27	0.1748		1.27	1.06	1.51	0.0095	*
**African American/Black**	**White**	3.87	3.28	4.57	1.92E-57	***	3.77	3.13	4.56	2.57E-43	***
**Hispanic/Latinx**		2.11	1.79	2.48	3.34E-19	***	1.65	1.35	2.03	1.26E-06	***
**Asian**		2.14	1.69	2.72	3.97E-10	***	1.20	0.84	1.73	0.3201	
**Multiracial/Other**		1.55	1.30	1.84	8.74E-07	***	1.60	1.29	1.98	2.16E-05	***
**1 Preexisting**	**0**	1.09	0.96	1.25	0.1766		1.03	0.87	1.22	0.7387	
**2 Preexisting**		0.95	0.82	1.10	0.4932		1.12	0.93	1.35	0.2247	
**3+ Preexisting**		1.15	0.98	1.35	0.0768		1.22	1.00	1.49	0.0481	*
**Preexisting Not Say**		1.34	0.79	2.27	0.2733		1.90	1.09	3.32	0.0246	*
**Parent**	**Not a Parent**	1.17	1.03	1.33	0.0127	*	1.52	1.30	1.78	2.84E-07	***
**Income 0-40K**	**100K+**	1.79	1.41	2.28	2.14E-06	***	1.62	1.21	2.17	0.0013	*
**Income 40-70K**		1.62	1.39	1.88	2.75E-10	***	1.56	1.30	1.88	2.51E-06	***
**Income 70-100K**		1.23	1.07	1.42	0.0049	*	1.13	0.94	1.36	0.1918	
**PopDensity 0-149**	**1000+**	1.27	1.09	1.49	0.0022	*	1.32	1.08	1.61	0.0071	*
**PopDensity 150-999**		1.19	1.06	1.34	0.0039	*	1.43	1.25	1.65	4.86E-07	***
**West**	**Northeast**	0.92	0.70	1.20	0.5409		1.17	0.84	1.61	0.3498	
**Midwest**		0.99	0.79	1.26	0.9603		1.29	0.97	1.70	0.0779	

**South**		1.07	0.85	1.35	0.5636		1.62	1.23	2.14	0.0006	**
**Answered before Pfizer EUA**	**After Pfizer EUA**	1.59	1.43	1.77	1.09E-17	***	1.33	1.16	1.51	2.65E-05	***
**Practiced Protective Behavior**	**Did Not Practice**	0.81	0.73	0.91	0.0003	**	0.73	0.64	0.84	1.34E-05	***
**State Cumulative Case Rate**		1.02	0.97	1.07	0.5056		1.00	0.94	1.06	0.9572	
**State Cumulative Death Rate**		1.14	0.93	1.41	0.2149		1.23	0.96	1.57	0.1046	
**Average Daily Pos Test Rate (Nov.)**		1.00	0.98	1.02	0.8418		0.99	0.96	1.01	0.2928	
**County % without HS Degree**		1.00	0.99	1.01	0.4943		1.01	0.99	1.02	0.4032	
**Received COVID Test**	**Never Received Test**	0.81	0.70	0.94	0.0060	*	0.72	0.60	0.88	0.0010	*

*: $p < 0.05$
**: $p < 0.001$
***: $p < 0.0001$

Supplementary Table 9: Receiving Test – Weighted logistic regression analysis for receiving a COVID-19 test.

Name	Reference Group	Probability of Receiving a COVID Test				
		OR	Lower 95% CI	Upper 95% CI	P-Value	Sig
**Female**	**Male**	0.92	0.84	1.01	0.0939	
**Age [18,30)**	**65+**	0.68	0.58	0.81	8.46E-06	***
**Age [30,45)**		0.82	0.71	0.93	0.0032	*
**Age [45,55)**		0.91	0.79	1.04	0.1670	
**Age [55,65)**		0.91	0.81	1.03	0.1455	
**Essential Healthcare**	**Nonessential**	2.12	1.91	2.36	9.19E-46	***
**Other Essential**		0.97	0.85	1.11	0.6502	
**Furloughed/Job Seeker**	**Employed**	0.93	0.79	1.10	0.4023	
**Unemployed**		0.84	0.75	0.94	0.0022	*
**African American/Black**	**White**	0.98	0.82	1.16	0.7966	
**Hispanic/Latinx**		0.88	0.75	1.03	0.1138	
**Asian**		0.96	0.77	1.21	0.7582	
**Multiracial/Other**		0.96	0.81	1.13	0.5965	
**1 Preexisting**	**0**	1.04	0.93	1.16	0.4715	
**2 Preexisting**		1.12	1.00	1.27	0.0597	
**3+ Preexisting**		1.48	1.31	1.68	6.57E-10	***
**Preexisting Not Say**		0.71	0.39	1.28	0.2550	
**Parent**	**Not a Parent**	0.95	0.87	1.05	0.3121	
**Income 0-40K**	**100K+**	1.06	0.86	1.32	0.5750	
**Income 40-70K**		0.99	0.89	1.11	0.9141	
**Income 70-100K**		1.02	0.92	1.13	0.7056	
**PopDensity 0-149**	**1000+**	0.78	0.67	0.90	0.0007	**
**PopDensity 150-999**		0.86	0.78	0.94	0.0012	*
**West**	**Northeast**	1.26	1.01	1.57	0.0438	*
**Midwest**		0.81	0.66	0.99	0.0394	*
**South**		1.17	0.97	1.42	0.0982	
**Answered before Pfizer EUA**	**After Pfizer EUA**	0.99	0.91	1.08	0.9030	
**Practiced Protective Behavior**	**Did Not Practice**	1.02	0.94	1.12	0.6097	

**State Cumulative Case Rate**	1.08	1.04	1.14	0.0005	**
**State Cumulative Death Rate**	1.40	1.18	1.68	0.0002	**
**Average Daily Pos Test Rate (Nov.)**	0.95	0.94	0.97	4.12E-06	***
**County % without HS Degree**	1.01	1.00	1.02	0.0240	*

*: p < 0.05
**: p < 0.001
***: p < 0.0001

Supplementary Table 10: IPW Analysis – IPW analysis utilizing the probability of receiving a COVID-19 test as an inverse probability weight.

		A IPW Trim (0.5,0.95) n=4,837					B IPW Trim (0.1,0.9) n=4,837				
**Variable**	**Reference Group**	**OR**	**Lower 95% CI**	**Upper 95% CI**	**P-Value**	**Sig**	**OR**	**Lower 95% CI**	**Upper 95% CI**	**P-Value**	**Sig**
**Female**	**Male**	1.62	1.24	2.10	0.0003	**	1.62	1.25	2.10	0.0003	**
**Age [18,30)**	**65+**	2.64	1.72	4.05	8.47E-06	***	2.68	1.77	4.07	3.32E-06	***
**Age [30,45)**		2.03	1.46	2.84	3.24E-05	***	2.01	1.45	2.79	2.86E-05	***
**Age [45,55)**		2.07	1.50	2.87	1.11E-05	***	2.04	1.49	2.81	1.07E-05	***
**Age [55,65)**		1.42	1.06	1.90	0.0193	*	1.42	1.06	1.89	0.0175	*
**Essential Healthcare**	**Nonessential**	1.28	1.04	1.59	0.0201	*	1.28	1.04	1.58	0.0207	*
**Other Essential**		1.16	0.87	1.53	0.3193		1.14	0.87	1.51	0.3434	
**Furloughed/Job Seeker**	**Employed**	1.20	0.85	1.70	0.2991		1.23	0.88	1.74	0.2279	
**Unemployed**		0.97	0.74	1.27	0.8379		0.98	0.75	1.27	0.8633	
**African American/Black**	**White**	4.01	2.87	5.61	6.26E-16	***	3.98	2.85	5.55	5.20E-16	***
**Hispanic/Latinx**		1.83	1.28	2.63	0.0009	**	1.79	1.26	2.53	0.0010	*
**Asian**		1.78	1.01	3.16	0.0474	*	1.76	1.00	3.12	0.0510	
**Multiracial/Other**		1.66	1.18	2.33	0.0037	*	1.62	1.16	2.28	0.0051	*
**1 Preexisting**	**0**	1.58	1.22	2.05	0.0006	**	1.59	1.23	2.04	0.0003	**
**2 Preexisting**		1.23	0.93	1.63	0.1503		1.26	0.96	1.66	0.0970	
**3+ Preexisting**		1.63	1.22	2.17	0.0009	**	1.64	1.24	2.17	0.0005	**
**Preexisting Not Say**		0.73	0.15	3.51	0.6933		0.79	0.17	3.72	0.7646	
**Parent**	**Not a Parent**	1.41	1.11	1.78	0.0044	*	1.40	1.12	1.76	0.0037	*
**Income 0-40K**	**100K+**	1.65	1.03	2.63	0.0365	*	1.66	1.05	2.63	0.0306	*
**Income 40-70K**		1.77	1.34	2.32	4.70E-05	***	1.75	1.34	2.29	4.75E-05	***
**Income 70-100K**		1.36	1.05	1.76	0.0191	*	1.34	1.04	1.72	0.0249	*
**PopDensity 0-149**	**1000+**	1.03	0.75	1.43	0.8506		1.03	0.75	1.42	0.8361	
**PopDensity 150-999**		1.18	0.96	1.46	0.1254		1.20	0.98	1.48	0.0810	
**West**	**Northeast**	0.65	0.39	1.09	0.1024		0.69	0.42	1.13	0.1373	
**Midwest**		0.94	0.59	1.49	0.7909		1.01	0.64	1.58	0.9771	
**South**		1.21	0.77	1.88	0.4078		1.23	0.80	1.89	0.3354	
**Answered before Pfizer EUA**	**After Pfizer EUA**	1.78	1.47	2.14	2.64E-09	***	1.78	1.48	2.14	1.36E-09	***

**Practiced Protective Behavior**	**Did Not Practice**	0.92	0.76	1.12	0.4286	0.93	0.77	1.13	0.4940
**State Cumulative Case Rate**		1.01	0.91	1.12	0.8462	1.01	0.91	1.11	0.9124
**State Cumulative Death Rate**		0.83	0.56	1.23	0.3537	0.85	0.58	1.25	0.4028
**Average Daily Pos Test Rate (Nov.)**		0.97	0.93	1.02	0.2570	0.97	0.93	1.02	0.2324
**County % without HS Degree**		1.02	1.00	1.04	0.0633	1.02	1.00	1.04	0.0630
**Tested Positive**	**Tested Negative**	1.25	0.79	1.96	0.3443	1.26	0.80	1.97	0.3154

*: p < 0.05
**: p < 0.001
***: p < 0.0001

Supplementary Table 11: Interim uptake analyses - Interim analyses for the weighting procedure of the vaccine uptake analysis. Left is the weighted model to predict if a user responded to the vacccine uptake question, while right is the weighted model for predicting if a user was offered a vaccine.

		A: Responded to Vaccine Uptake Question (n = 23,782)					B: Offered Vaccine (n = 18,928)				
**Variable**	**Reference Group**	**OR**	**Lower 95% CI**	**Upper 95% CI**	**P-Value**	**Sig**	**OR**	**Lower 95% CI**	**Upper 95% CI**	**P-Value**	**Sig**
**Female**	**Male**	0.92	0.86	0.98	0.0142	*	1.09	1.00	1.20	0.0615	
**Age [18,30)**	**65+**	0.35	0.31	0.40	4.06E-69	***	0.05	0.04	0.06	5.78E-264	***
**Age [30,45)**		0.45	0.41	0.50	2.90E-57	***	0.09	0.08	0.11	8.67E-215	***
**Age [45,55)**		0.65	0.59	0.72	6.45E-17	***	0.15	0.13	0.17	2.06E-139	***
**Age [55,65)**		0.88	0.81	0.97	0.0079	*	0.26	0.23	0.30	3.56E-80	***
**Essential Healthcare**	**Nonessential**	0.84	0.77	0.92	0.0002	**	3.90	3.30	4.60	1.06E-57	***
**Other Essential**		0.95	0.87	1.05	0.3164		0.72	0.65	0.80	3.23E-09	***
**Furloughed/Job Seeker**	**Employed**	0.93	0.83	1.05	0.2525		0.61	0.53	0.70	1.96E-12	***
**Unemployed**		1.18	1.09	1.28	6.29E-05	***	0.72	0.65	0.80	1.51E-10	***
**African American/Black**	**White**	1.32	1.16	1.50	1.85E-05	***	0.96	0.80	1.14	0.6345	
**Hispanic/Latinx**		1.00	0.89	1.12	0.9905		1.00	0.85	1.17	0.9727	
**Asian**		1.04	0.89	1.22	0.5928		0.98	0.77	1.24	0.8396	
**Multiracial/Other**		1.22	1.09	1.37	0.0007	**	0.80	0.70	0.92	0.0016	*
**1 Preexisting**	**0**	0.97	0.90	1.05	0.4792		1.03	0.93	1.13	0.5779	
**2 Preexisting**		0.93	0.86	1.02	0.1189		1.13	1.01	1.25	0.0335	*
**3+ Preexisting**		1.04	0.95	1.15	0.3825		1.01	0.90	1.14	0.8321	
**Preexisting Not Say**		1.14	0.83	1.56	0.4264		1.06	0.74	1.51	0.7608	
**Parent**	**Not a Parent**	0.85	0.79	0.91	6.41E-06	***	0.89	0.82	0.98	0.0120	*
**Income 0-40K**	**100K+**	1.07	0.91	1.24	0.4178		0.98	0.80	1.20	0.8425	
**Income 40-70K**		1.06	0.97	1.15	0.2059		0.98	0.88	1.09	0.6805	
**Income 70-100K**		1.10	1.02	1.19	0.0179	*	0.99	0.90	1.09	0.8639	
**PopDensity 0-149**	**1000+**	0.83	0.75	0.92	0.0002	**	0.91	0.80	1.04	0.1681	
**PopDensity 150-999**		0.85	0.80	0.91	4.20E-06	***	1.02	0.93	1.11	0.6911	
**West**	**Northeast**	1.11	0.95	1.30	0.1842		0.96	0.79	1.17	0.6803	
**Midwest**		1.05	0.92	1.21	0.470		0.91	0.77	1.09	0.3125	
**South**		1.02	0.89	1.17	0.7614		0.87	0.73	1.04	0.1201	
**Practiced Protective Behavior**	**Did Not Practice**	0.62	0.58	0.66	1.35E-51	***	0.66	0.61	0.72	3.38E-24	***

**State Cumulative Case Rate**		0.98	0.95	1.01	0.2732	1.01	0.97	1.05	0.720
**State Cumulative Death Rate**		1.09	0.96	1.23	0.1729	1.03	0.88	1.21	0.7137
**Average Daily Pos Test Rate (Nov.)**		1.01	0.99	1.02	0.2767	1.00	0.98	1.01	0.6282
**County % without HS Degree**		0.99	0.99	1.00	0.1606	1.01	1.00	1.02	0.1056
**Received COVID Test**	**Never Received Test**	0.94	0.86	1.02	0.1488	1.23	1.09	1.38	0.0005 **
**Vaccine Acceptance - Unlikely**	**Vaccine Acceptance - Likely**	0.84	0.75	0.94	0.0029 *	0.51	0.44	0.59	2.53E-19 ***
**Vaccine Acceptance - Undecided**	**Vaccine Acceptance - Likely**	0.91	0.83	0.99	0.0363 *	0.57	0.51	0.64	1.33E-22 ***

*: $p < 0.05$
**: $p < 0.001$
***: $p < 0.0001$

Supplementary Table 12: Vaccination Uptake Analyses - Unweighted model for predicting vaccine uptake (left) and a weighted model for predicting vaccine uptake accounting for non-response bias and biases associated with being offered a vaccine.

		A					B				
		Unweighted Uptake Analysis (n = 18,928)					Weighted Uptake Analysis (n = 18,928)				
Variable	Reference Group	OR	Lower 95% CI	Upper 95% CI	P-Value	Sig	OR	Lower 95% CI	Upper 95% CI	P-Value	Sig
**Female**	**Male**	0.74	0.52	1.03	0.0808		0.90	0.60	1.33	0.5868	
**Age [18,30)**	**65+**	0.13	0.08	0.20	2.41E-18	***	0.10	0.06	0.18	1.43E-16	***
**Age [30,45)**		0.23	0.16	0.34	7.06E-14	***	0.20	0.13	0.31	1.29E-13	***
**Age [45,55)**		0.40	0.27	0.59	3.94E-06	***	0.38	0.25	0.57	3.68E-06	***
**Age [55,65)**		0.57	0.39	0.82	0.0025	*	0.55	0.37	0.81	0.0029	*
**Essential Healthcare**	**Nonessential**	0.69	0.53	0.92	0.0107	*	0.74	0.54	1.01	0.0540	
**Other Essential**		0.65	0.48	0.90	0.0077	*	0.64	0.44	0.92	0.0162	*
**Furloughed/Job Seeker**	**Employed**	0.92	0.61	1.41	0.6821		0.96	0.60	1.56	0.8841	
**Unemployed**		1.00	0.74	1.36	0.9874		0.86	0.61	1.22	0.3991	
**African American/Black**	**White**	0.74	0.52	1.08	0.1129		0.58	0.38	0.91	0.0165	*
**Hispanic/Latinx**		0.80	0.54	1.21	0.2790		1.07	0.62	1.84	0.8080	
**Asian**		2.23	0.86	7.69	0.1432		2.45	0.78	7.73	0.1266	
**Multiracial/Other**		0.79	0.53	1.20	0.2465		0.72	0.43	1.21	0.2164	
**1 Preexisting**	**0**	0.85	0.63	1.14	0.2869		0.99	0.69	1.43	0.9620	
**2 Preexisting**		0.88	0.64	1.21	0.4216		1.00	0.69	1.46	0.9995	
**3+ Preexisting**		0.87	0.62	1.22	0.4198		1.05	0.70	1.60	0.8072	
**Preexisting Not Say**		0.41	0.17	1.11	0.0561		0.95	0.22	4.14	0.9412	
**Parent**	**Not a Parent**	0.78	0.60	1.01	0.0584		0.63	0.45	0.89	0.0086	*
**Income 0-40K**	**100K+**	0.81	0.46	1.44	0.4642		0.79	0.39	1.62	0.5221	
**Income 40-70K**		0.53	0.37	0.76	0.0007	**	0.56	0.37	0.85	0.0066	*
**Income 70-100K**		0.64	0.44	0.91	0.0139	*	0.63	0.42	0.96	0.0316	*
**PopDensity 0-149**	**1000+**	0.48	0.35	0.67	1.82E-05	***	0.53	0.34	0.82	0.0049	*
**PopDensity 150-999**		0.79	0.61	1.01	0.0581		0.81	0.59	1.11	0.1841	
**West**	**Northeast**	0.87	0.50	1.49	0.6039		0.95	0.50	1.79	0.8640	
**Midwest**		0.75	0.46	1.22	0.2380		0.73	0.43	1.23	0.2372	
**South**		0.62	0.38	1.01	0.0563		0.69	0.40	1.17	0.1669	
**Practiced Protective Behavior**	**Did Not Practice**	1.17	0.91	1.53	0.2209		1.06	0.79	1.41	0.7170	
**State Cumulative Case Rate**		0.95	0.85	1.06	0.3754		0.99	0.86	1.13	0.8526	
**State Cumulative Death Rate**		0.83	0.54	1.28	0.4071		0.90	0.55	1.46	0.6601	
**Average Daily Pos Test Rate (Nov.)**		0.98	0.94	1.02	0.3269		0.97	0.92	1.02	0.1763	

**County % without HS Degree**		1.00	0.97	1.02	0.8621		0.99	0.96	1.02	0.6456	
**Received COVID Test**	**Never Received Test**	1.00	0.73	1.39	0.9845		1.05	0.75	1.49	0.7635	
**Vaccine Acceptance - Unlikely**	**Vaccine Acceptance - Likely**	0.02	0.01	0.02	2.25E-183	***	0.02	0.01	0.03	2.07E-114	***
**Vaccine Acceptance - Undecided**	**Vaccine Acceptance - Likely**	0.08	0.06	0.11	1.22E-67	***	0.08	0.06	0.12	1.06E-39	***

*: $p < 0.05$
**: $p < 0.001$
***: $p < 0.0001$

Supplementary Table 13: Reluctant User Analysis - Weighted vaccine uptake model for only users that responded as "reluctant" to the vaccine intent question.

		Vaccine Uptake Analysis for Reluctant Users (n = 2,520)				
**Variable**	**Reference Group**	**OR**	**Lower 95% CI**	**Upper 95% CI**	**P-Value**	**Sig**
**Female**	**Male**	0.68	0.44	1.06	0.0894	
**Age [18,30)**	**65+**	0.14	0.07	0.25	5.48E-10	***
**Age [30,45)**		0.27	0.16	0.45	4.79E-07	***
**Age [45,55)**		0.40	0.24	0.66	0.0003	**
**Age [55,65)**		0.60	0.37	0.99	0.0460	*
**Essential Healthcare**	**Nonessential**	0.66	0.47	0.94	0.0222	*
**Other Essential**		0.65	0.44	0.97	0.0336	*
**Furloughed/Job Seeker**	**Employed**	1.18	0.69	2.01	0.5501	
**Unemployed**		1.11	0.74	1.66	0.6231	
**African American/Black**	**White**	0.76	0.50	1.15	0.1908	
**Hispanic/Latinx**		0.98	0.57	1.68	0.9379	
**Asian**		3.18	0.70	14.42	0.1337	
**Multiracial/Other**		0.80	0.46	1.38	0.4241	
**1 Preexisting**	**0**	0.97	0.67	1.39	0.8504	
**2 Preexisting**		0.87	0.59	1.29	0.4989	
**3+ Preexisting**		0.94	0.61	1.44	0.7672	
**Preexisting Not Say**		2.34	0.61	8.98	0.2158	
**Parent**	**Not a Parent**	0.79	0.57	1.10	0.1565	
**Income 0-40K**	**100K+**	1.15	0.55	2.38	0.7143	
**Income 40-70K**		0.60	0.39	0.90	0.0149	*
**Income 70-100K**		0.80	0.52	1.23	0.3099	
**PopDensity 0-149**	**1000+**	0.48	0.31	0.75	0.0012	*
**PopDensity 150-999**		0.79	0.58	1.08	0.1428	
**West**	**Northeast**	0.76	0.39	1.51	0.4392	
**Midwest**		0.75	0.41	1.37	0.3510	
**South**		0.66	0.36	1.20	0.1764	
**Practiced Protective Behavior**	**Did Not Practice**	0.91	0.67	1.25	0.5592	
**State Cumulative Case Rate**		0.93	0.80	1.08	0.3527	
**State Cumulative Death Rate**		0.79	0.46	1.36	0.3954	
**Average Daily Pos Test Rate (Nov.)**		0.99	0.93	1.04	0.6154	
**County % without HS Degree**		0.99	0.96	1.03	0.6810	
**Received COVID Test**	**Never Received Test**	0.90	0.60	1.35	0.6181	
**Vaccine Acceptance - Undecided**	**Vaccine Acceptance - Unlikely**	4.57	3.47	6.03	2.26E-26	***

*: $p < 0.05$
**: $p < 0.001$
***: $p < 0.0001$
